# Supplementary material for: Common and distinct neural representations of aversive somatic and visceral stimulation in healthy individuals
Source: Nat Commun. 2020 Nov 23;11:5939. doi: 10.1038/s41467-020-19688-8 (PMC7684294; doi:10.1038/s41467-020-19688-8)
Supplement: Supplementary file 1 — Supplementary Information [file 41467_2020_19688_MOESM1_ESM.pdf]

# Common and distinct neural representations of aversive somatic and visceral stimulation in healthy individuals

## Supplementary Information

## Supplementary Methods

*Participants*

The studies included a total of 165 healthy subjects. **Study 1** (previously unpublished) (gastric pain) included 15 subjects (10 women) with a mean (±SD) age of 31.9±8.8 years. **Study 2**[1] (rectal discomfort) included 15 subjects (9 women) with a mean age of 29.5±10.5 years. **Study 3**[2] (rectal discomfort) included 29 subjects (15 women) with a mean age of 22.5±2.8 years. **Study 4**[3] (vulvar pain) included 15 subjects (15 women) with a mean age of 23.2±1.6 years. **Study 5**[4] (esophageal pain) included 30 subjects (14 women) with a mean age of 30.4±8.7 years. **Study 6** (cutaneous thermal pain) included 28 subjects (10 women) with a mean age of 25.2±7.4 years[5]. **Study 7** (cutaneous thermal pain) included 33 subjects (22 women) with a mean age of 27.9±9.0 years[6]. Excluding Study 4 (vulvar stimulation in females) the combined sample is 54.6% female (range = [46.7% 66.7%], SD = 12.2%). A 2 (female/male) x 6 (study) $\chi^2$ test for sex differences across studies did not reveal evidence for significant differences across studies ($\chi^2(5) = 7.76$, $p > 0.15$), suggesting that differences in brain activity between different stimulation types cannot simply be interpreted as reflecting differences in sex.

All studies were approved by the respective medical ethics committees of the institutions where the studies were performed (Study 1 and 4: University of Leuven, Belgium; Study 2: Université de Grenoble Joseph Fourier, France; Study 3: Tohoku University, Sendai, Japan; Study 5: King's

College London, UK; Study 6: University of Colorado, Boulder, USA; Study 7: Columbia University, New York, USA). Only healthy volunteers, recruited by means of local advertisement, without any medical or psychiatric disorders and not currently taking any medication affecting the central nervous system were included. All subjects provided written informed consent prior to being included in the studies at all sites.

*Pain/discomfort threshold determination and stimulation procedures*

**Study 1:** After an overnight fast of at least 12h, a double-lumen polyvinyl tube (Salem sump tube 14 Ch; Sherwood Medical, Petit Rechain, Belgium) with a finely folded adherent polyethylene bag (maximal volume 1200ml) was intubated through the mouth. The tube was then secured to the subject's chin with adhesive tape and connected to a barostat device (GandJ Electronics Inc., Toronto, Canada). The subject was then positioned in the same position as later in the MR scanner, i.e. lying down in supine position. In order to unfold the bag, it was inflated with a fixed volume of 300 mL of air for 5 min and then deflated completely. Next, the minimal distending pressure (MDP) - i.e. the lowest pressure level needed to equilibrate the intra-abdominal pressure - was determined by increasing the intra-bag pressure by 1 mm Hg every 3 minutes until a volume of 30 ml was reached. Subsequently, the individual pain threshold for gastric balloon distension was determined by means of ascending method of limits (AML) (starting intra-bag pressure: MDP+2 mmHg, increments: 2 mmHg, duration of each step: 2 minutes). At the end of each distension step, subjects were asked to rate their perceived gastric pain using a visual analogue scale (VAS) from 0 to 100 (0 = "no pain" and 100 = "intolerable pain"). The AML stopped when the subject rated their gastric pain $\geq 40$ (moderate pain). The average intra-bag volume during the last distension step of the AML was used as painful gastric distension stimulus in the fMRI sessions. The reproducibility of the rating for the identified

volume was subsequently verified in a volumetric distention sequence that was identical to the distention sequence used during the trials in the fMRI experiment (i.e., an ultra-rapid volumetric distention to the pain threshold using a custom-made function on the barostat and deflation after 30 s). No information was given to the subjects regarding the distention volume and its consistency throughout the experiment.

**Studies 2 and 3:** On the day before the fMRI scan, subjects were given low residue meals, 17 g (13.6%) of magnesium citrate, 75 mg of sodium picosulphate, and 24 mg of sennoside A and B to cleanse the colon. Individual rectal discomfort threshold was defined prior to the fMRI scanning session (on the same day). For this purpose, a polyethylene bag, tightly fixed at both ends to a catheter, was inserted into the rectum with the distal end of the bag positioned 10 cm from the anal verge and taped in place. The maximal volume of the bag was 700 ml, and the maximal diameter and length of the bag at full inflation was 10 cm. Rectal distensions were performed with a pressure controlled barostat system (G&J Electronics Inc., Toronto, Canada). The subject was positioned on a bed in a quiet preparation room in a comfortable supine position. After a 10-min acclimatization period, the balloon was inflated with a barostat using staircase distensions with pressure increments of 5 mmHg. After each step, there was a rating period of 30 s for pain, discomfort, and urge to defecate with a verbal numerical rating scale from 0 to 100 (0 = “none” and 100 = “intolerable discomfort, pain or urge to defecate”). The retained discomfort threshold was the volume eliciting a 40-60% discomfort (severe discomfort). This intensity was used rather than pain threshold as 1) subjects had to tolerate the intensity for the entire duration of the fMRI examination, and 2) our pilot study, as well as previous studies[7] showed that the majority of the healthy volunteers reported severe urge or a corresponding severe discomfort to the highest level of stimulation by the barostat, before/without reporting

pain. Therefore, and given the safety limit of the barostat (maximal pressure of 50 mmHg), we could not use distention up to pain thresholds as our stimulus. The reproducibility of the rating for the identified volume was subsequently verified in a volumetric distention sequence that was identical to the distention sequence used during the trials in the fMRI experiment (i.e., an ultra-rapid volumetric distention to the discomfort threshold using a custom-made function on the barostat and deflation after 18 s). No information was given to the subjects regarding the distention volume and its consistency throughout the experiment.

**Study 4:** The vulvalgesiometer consists of a set of devices developed to standardize assessment of vulvar pressure-pain thresholds and has been used in multiple studies[8-12]. In this study, we used a modified version of the vulvalgesiometer, developed in collaboration with the Medical Instrumentation Department of the University Hospitals Leuven[3]. More specifically, the modified devices are longer, the tail-end is revolving so that pressure levels can be fixed, and all devices are made from a material that can be sterilized. These adaptations enabled us to use the vulvalgesiometer in a more accurate, user-friendly and hygienic way. In order to prevent injury at the vulvar vestibule during consecutive painful stimulation, the maximum applied pressure in this study was 400g - and not 500g as previously suggested[11]. The individual vulvar pressure-pain threshold was determined according to the standardized procedure described by Pukall[11]. Briefly, the cotton-swab tip of the device was applied to the genital area, held for 1-2 seconds on the vestibule at 5 o'clock and then removed. The lowest pressure level (3g) was applied first and was followed by higher pressure levels (5g, 10g, 15g, …, 400g). At the end of each stimulation, there was a rest period of 10s in which participants were asked to rate the intensity of vulvar pain on a VAS (ranging from 0; 'no pain' to 10; 'worst pain ever'). The gynecologist stopped applying the next pressure level when the participant reported a VAS score for pain intensity of

minimum 4 (without the participant being informed about this cut-off criterion). The reproducibility of the rating for the identified pressure was subsequently verified in a sequence that was identical to the sequence used during the trials in the fMRI experiment (12 second blocks, consisting of 3 stimulations of 4 seconds each). No information was given to the subjects regarding the pressure level and its consistency throughout the experiment.

**Study 5:** Painful phasic esophageal stimulation was delivered by distending a 2-cm-long silicone balloon with air, as previously described[4], with each distension lasting 1 s. The balloon was mounted 15 cm from the tip of a 4-mm-diameter standard manometry catheter (commercially available esophageal distention catheter; Sandhill Scientific, Oxford, England). The balloon was passed transnasally into the esophagus, and the center of the balloon was positioned 5 cm proximal to the lower esophageal sphincter. To determine pain toleration threshold in each volunteer, the volume of distension was increased in steps of 1 ml (from zero) until the subjects reached the point at which they could no longer tolerate an increase in stimulus. The pain toleration threshold level was recorded and used as the level of stimulation during scanning.

**Study 6:** Thermal stimulation was applied to the volar surface of the left forearm and dorsal surface of the left foot. Each stimulation lasted a total of 11 seconds with a 2 second ramp-up, 7 seconds at the peak target temperature, and a 2 second ramp-down. Three pseudo-randomly assigned temperature levels (level 1: 46°C, level 2: 47°C, level 3: 48°C) were applied to four different locations on both the forearm and foot.

**Study 7:** Thermal stimulation was delivered to the left volar forearm at different locations that alternated between runs. Each stimulus lasted 12.5 seconds: 3 second ramp-up, 7.5 seconds at the target temperature, and 2 second ramp-down. Heat was administered at six temperatures (level 1: 44.3°C, level 2: 45.3°C, level 3: 46.3°C, level 4: 47.3°C, level 5: 48.3°C, level 6: 49.3°C).

6

# VISCERAL AND SOMATIC PAIN

115

*fMRI task design*

**General design, studies 1-4:** Each trial consisted of a cued pain anticipation period, followed by a pain induction period and a rating and rest period. The fMRI session consisted of six runs, comprising of 12 trials each, with 2 minutes rest between the runs. The duration of the anticipation period varied randomly between 9 to 18 seconds and the anticipation was triggered by visual cues (symbol presented for 3 seconds, followed by a fixation point until the end of the pain period): an exclamation point ("!") indicated a 100% chance that pain would be induced during the subsequent pain period (certain condition); a zero ("0") indicated that no pain would be induced (safe condition); and a question mark ("?") indicated a 50% chance that pain would be induced (uncertain condition). When applicable, the anticipation period was followed by a pain induction period, the length of which varied between studies due to the different nature of the stimuli (see below). Immediately after each pain induction period, a rating and rest period followed which lasted for 24 seconds in total. This last part of the trial contained retrospective ratings about the fear of pain during the anticipation period and the pain intensity experienced during the painful stimulation. The VAS ratings (ranging from 0 to 10 with 0 = 'no (fear of) pain', and 10 = 'worst (fear of) pain ever') were presented on a screen and participants had to move a cursor (presented on the screen with a randomized starting position) interactively to the left or right by two keys on a button response box in order to give their ratings. Each of the three anticipation conditions (i.e., safe, uncertain and certain) was repeated 24 times in total. All conditions were randomized within each run, with the constraint that each condition was presented 4 times in each run. The sequence of stimuli during the fMRI experiment was controlled by Presentation® software (NeuroBehavioral Systems, San Francisco, California) installed on a computer connected to the MRI scanner, the screen and the response button box

(and a projector and headphones used to notify the gynaecologist about the timing of stimulation in the case of Study 4, see below).

**Study 1:** The length of the pain induction period was 30 seconds (including an inflation and a plateau phase) as pilot studies indicated this was the minimum amount of time needed to reach the high balloon volumes needed to induce gastric pain (with a custom-made ultra-rapid volumetric distension function on the barostat). The relative duration of the ramp up and the plateau phase varied slightly depending on the individually titrated volume threshold, with the ramp up phase taking approximately 20 seconds, leaving approximately 10 seconds for the plateau phase at pain threshold. Deflation started 30 seconds after the start of inflation and was performed during the ratings and rest period. Balloon inflations were controlled by the barostat device which was triggered by the Presentation program controlling stimulus delivery and collection of ratings.

**Studies 2 and 3:** The length of the discomfort induction period was 18 seconds as this was sufficient to reach the balloon volumes needed to induce rectal discomfort, with the ramp up phase again taking approximately 2/3 of the time, leaving about 1/3 for the plateau phase at discomfort threshold. Stimuli were delivered in the same way as in Study 1.

**Study 4:** The length of the pain induction period was 12 seconds, consisting of 3 stimulations of 4 seconds each. The female gynaecologist who was involved in the screening procedure performed the painful stimulation during all scan sessions by means of the modified vulvalgesiometer. For this purpose, the gynaecologist simultaneously received 3 visual cues (on the outer housing of the scanner) and auditory cues (via the headphone) of 4 seconds each in order to time the induction of pain correctly. These cues were not visible or audible for the participant in the scanner.

**Study 5:** Balloon distention of the distal esophagus (1-s duration) was delivered at pain toleration threshold; timing of each stimulus was controlled using custom-made software synchronized with scanner acquisition. Each trial commenced with the presentation of a visual warning cue (a coloured square) followed by a painful esophageal distension stimulus, which was in turn followed by a second visual cue (a differently coloured square) which signalled a “safe” condition during which there was no risk of stimulation. Following each stimulation, a visual analog scale (VAS) was presented and subjects rated the intensity of stimulation using an MRI-compatible button box, positioned in the right hand, to move a cursor from 0 (no sensation) to 100 (maximal pain). The timing of each condition (anticipation and pain) of the event-related design was pseudo-randomized and jittered to the TR (repetition time) to avoid habituation and enable a representative sample of the brain response during each condition; the anticipation phase signalled the start of a new trial and lasted between 3 and 12 s, whereas the “safe” phase lasted between 28 and 35 s including the VAS, which commenced 9–15 s after the onset of painful stimulation. The safe phase included a null event that served as the baseline condition. The use of colored squares as signals was also pseudo-randomized such that half the subjects received the blue square as a warning signal and yellow as safe and the other half received the yellow square as a warning and blue as safe. The functional imaging run consisted of 20 trials incorporating 60 events: 20 periods of anticipation, 20 painful esophageal distensions, and 20 null events (rest/baseline).

**Study 6:** fMRI data acquisition of thermal stimulation included 9 functional runs. Each experimental run contained 9 trials. Experimental trials began with a 2s cue followed by a pseudo-random jitter lasting 5, 7, or 11 seconds to isolate brain responses to the cue and thermal stimulation. Thermal stimulation followed for 11 seconds. After another jittered fixation lasting

2, 6 or 14 seconds, participants had 4 seconds to rate their experience of the stimulus on a visual analog scale using a trackball.

**Study 7:** fMRI data acquisition included 10 functional runs. Runs 1, 2, 4, 8 and 9 included 11 stimulations at each of levels 1-5, for a total of 55 stimuli. On runs 5-6 temperatures were increased one degree, with 4 stimuli at each of levels 2-6. Each trial consisted of a stimulus (12.5 seconds), a 4.5-8.5 second delay, a 4 second painful/nonpainful decision period, a 7 second continuous warmth or pain rating period, and 23-27 seconds of rest.

*MRI data acquisition*

**Studies 1 and 4:** Data were acquired on a Philips Achieva 3.0 Tesla scanner (Philips Medical Systems, Best, the Netherlands) with a 32-channel head coil. Functional images were obtained using an echo-planar imaging (EPI) sequence with blood oxygen level-dependent (BOLD) contrast (TR/TE=3000/30 ms, voxel size=2.50 × 2.50 × 2.50 $mm^3$, flip angle 90°, 48 slices of 2.5 mm thick) covering the whole brain including the cerebellum. Stimulus presentation and behavioral data acquisition were controlled using Presentation. A high resolution structural scan of the whole brain was acquired using a 3-dimensional T1-weighted Magnetization Prepared Rapid Acquisition Gradient Echo (3D-MPRAGE) sequence (TR/TE=9.6/4.6 ms, voxel size 0.98 × 0.98 ×1.20 $mm^3$).

**Study 2:** Data were acquired on a Philips Achieva 3.0 Tesla MRI scanner (Philips Medical Systems, Best, Netherlands) with a 32-channel head coil. Functional images were obtained with a gradient EPI sequence (TR/TE=3000/30 ms; flip angle 80°; 52 slices of 3 mm thick; voxel size 2.75 × 2.75 × 3 $mm^3$) covering the whole brain including the cerebellum. Stimulus presentation and behavioral data acquisition were controlled using Presentation. A high resolution structural

scan of the whole brain was acquired using a 3D-MPRAGE sequence (TR/TE=25/3.9 ms, voxel size 0.89 × 0.89 × 1.2 $mm^3$).

**Study 3:** Data were acquired on a 3T SIEMENS MAGNETOM TrioTim scanner with a 32 channel head coil. Functional images were collected using an EPI sequence with BOLD contrast (TR/TE = 3000/30 ms, voxel size = 2.5 × 2.5 × 2.5 $mm^3$, flip angle 90°, 50 slices) covering the whole brain including the cerebellum. Stimulus presentation and behavioral data acquisition were controlled using Presentation. A high resolution structural scan of the whole brain was acquired using a 3D-MPRAGE sequence (TR/TE = 2800/2.8 ms, voxel size 1.0 × 1.0 × 1.1 $mm^3$).

**Study 5:** Data were collected on a General Electric Signa Excite II 3.0 T scanner During fMRI, a total of 480 T2* weighted EPI images (40×3-mm slices, 0.3 interslice gap, TE 30 ms, TR 2,500 ms, flip angle 80°, matrix size 642, voxel size 3.75 × 3.75 × 3.3 $mm^3$) were collected. Stimulus presentation and behavioral data acquisition were controlled using custom-made Visual Basic software. A high-resolution Gradient Echo structural scan (TR 3,000 ms, TE = 30 ms, 43×3-mm slices, 0.3 interslice gap, flip angle 90°, matrix size 1282, voxel size 1.875 × 1.875 × 3.3 mm)was acquired.

**Study 6:** Functional images were acquired on a 3T Siemens Trio scanner with an EPI sequence (TR = 1300 ms, TE = 25 ms, field of view = 220 mm, 64 × 64 matrix, 3.4 × 3.4 × 3.4 $mm^3$ voxels, 26 interleaved slices with ascending acquisition, parallel imaging with an iPAT acceleration of 2) covering the whole brain. Stimulus presentation and behavioral data acquisition were controlled using MATLAB. Structural images were acquired using a high-resolution T1 spoiled gradient recall (SPGR) sequence.

**Study 7:** Data were acquired on a 3T Philips Achieva TX scanner. Functional EPI images were acquired with TR = 2000 ms, TE = 20 ms, field of view = 224 mm, 64 × 64 matrix, 3 × 3 × 3 $mm^3$ voxels, 42 interleaved slices, parallel imaging, SENSE factor 1.5 covering the whole brain. Stimulus presentation and data acquisition were controlled using E-Prime. Structural images were acquired using a high-resolution T1 SPGR sequence.

*fMRI data preprocessing*

**Studies 1-5:** Data were pre-processed and analyzed using SPM8 software (Wellcome Trust Centre for Neuroimaging, UCL). Pre-processing included spatial realignment to correct for small movements, slice-timing, co-registration of the functional images and the structural image, segmentation of the structural image and warping to Montreal Neurological Institute (MNI) space based on the structural image and based on the transformation obtained during the segmentation step. The warping parameters were applied to the functional images and finally, the warped functional images were smoothed with a Gaussian isotropic kernel with 8-mm full width at half maximum.

**Study 6:** Preprocessing was performed with SPM8 (Wellcome Trust Centre for Neuroimaging, London, UK). Structural images were then co-registered to the first functional image for each participant with an iterative procedure of automated registration using mutual information from the co-registration in SPM8. Next, images were normalized to MNI space and interpolated to 2 × 2 × 2 mm voxels. Prior to preprocessing of functional images, global outlier time points were identified by computing both the mean and the standard deviation (across voxels) of values for each image for all slices. The output of this procedure was later used as nuisance regressors in the first level models. Functional images were corrected for slice-acquisition-timing and realigned using SPM8. Next, they were warped to SPM8's normative atlas using parameters estimated from the co-registered structural images, interpolated to 2 × 2 × 2 mm voxels, and smoothed with an 8 mm FWHM Gaussian kernel.

**Study 7:** Structural T1-weighted images were coregisted to the mean functional image for each subject using SPM's iterative mutual information-based algorithm. Coregistered structural images were normalized to MNI space using the integrated segmentation and normalization

algorithm using the default parameters (7 × 8 × 7 nonlinear basis functions) and resampled to 2 × 2 × 2 mm voxels.

Functional images underwent outlier detection and removal, in which time points flagged as outliers were imputed with the voxel's run mean. Data across the entire run were winsorized to three standard deviations. Slice time correction was applied to correct for differences in the acquisition time for each slice. A 6-parameter registration to the mean functional image was applied to correct for head movement during scanning. Time series data for each voxel were converted to percent signal change based on a spatially smoothed baseline time series (16 mm FWHM). Finally, warping parameters estimated from coregistered structural images were applied, and functional images were upsampled to 2 × 2 × 2 mm voxels.

*fMRI data analysis*

**Studies 1-4:** Analyses were conducted with a combined event (anticipation period) and block (pain period) design using the general linear model (GLM) implemented in SPM8. Each pain condition was modelled as a box-car stimulus function convolved with the canonical hemodynamic response function and entered into a standard generalized linear model that includes high-pass filtering with a cut-off frequency of 1/128 s to remove low-frequency drifts in the signals. There were 7 regressors of interest corresponding to 3 anticipation (certain, uncertain, and safe) and 4 pain (certain$_{pain}$, uncertain$_{pain}$, safe$_{nopain}$, uncertain$_{nopain}$) conditions, and realignement parameters and run were included as regressors of no interest. For the present study, only the responses to pain induction following the certain anticipation cue (relative to the no pain period following the safe anticipation cue) are used (i.e. the contrast certain$_{pain}$ – safe$_{nopain}$ was estimated for each subject). The 3 anticipation conditions, the uncertain$_{pain}$ and uncertain$_{nopain}$ pain conditions, and the rating and rest period were included in the design matrix

but not used in any contrasts as they are not required to answer the research questions formulated in this paper.

**Study 5:** Analyses were conducted with an event-related design using the generalized linear model implemented in SPM8. The 20 trials consisting of anticipation, painful esophageal distension, and null events (rest/baseline) were divided into 4 periods (each consisting of 5 trials). To create regressors of interest, each event was modelled by convolving delta functions at onset of presentation of the anticipatory cue or painful esophageal stimulation with the canonical hemodynamic response function. There were 3 regressors of interest (anticipation, painful esophageal stimulation, and rest) and 7 regressors of no interest (rating epoch and 6 realignment parameters). A high-pass filter of 128s was used to remove low-frequency drifts in the signals. The contrast painful esophageal distention compared to rest was computed for each individual and used in this paper.

**Study 6:** First-level GLM analyses were conducted in SPM8. The first 6 volumes of each run were discarded, and the nine experimental runs were concatenated for each participant (the first two conditioning runs were excluded). Boxcar regressors, convolved with the canonical hemodynamic response function, were constructed to model periods for the 2 second cue presentation, the 5, 7, or 11 second variable pre-stimulus fixation period, the 11 second thermal stimulation (9 levels), and 4 second rating periods. The fixation cross epoch was used as an implicit baseline. A high-pass filter of 224s was used. The contrast used for the present study included a comparison of high stimulation levels (48°C) versus baseline, collapsed across body site and cue.

**Study 7:** First-level GLM analyses were conducted in SPM8. The design matrix included a set of 7 regressors constructed for conditions of interest by convolving epochs lasting the duration of

each event with the canonical hemodynamic response function. Nuisance regressors were included for each run. They comprised a constant term, dummy regressors for estimated outlier images from preprocessing, and 24 movement-related covariates based on estimated movement during realignment, including 6 mean-centered motion parameter estimates, their squared values, their successive differences, and squared successive differences. For the present study, the contrast of interest was a comparison of high (the average of 48°C and 49°C) versus low (the average of 44°C and 45°C) levels of stimulation.

## Supplementary Results

Correlation patterns of brain responses to different types of visceral and somatic stimulation with resting-state networks

To further test if pain representations differ at a broad spatial scale, we assessed whether brain responses to visceral and somatic stimulation were differentially correlated with 7 canonical resting-state networks [13]. As shown in Table S12 and briefly summarized below, different patterns of point-biserial correlations were found across individual stimulation types. More specifically, activations during all 4 visceral stimulation studies were reliably and positively correlated with the frontoparietal network. Conversely, activation during painful vulvar stimulation (Study 4) did not exhibit this positive relationship and the responses to thermal stimulation (Study 6 and 7) showed negative correlations with the frontoparietal network that did not reach significance after correction for multiple comparisons. Furthermore, brain activation elicited by somatic and visceral stimulation also differed in their similarity to the somatomotor network. Somatic studies reliably exhibited positive correlations with this network under both vulvar (Study 4) and thermal (Study 6 and 7) stimulation. Correlations between activation during visceral stimulation and the somatomotor network varied depending on stimulus type. More

specifically, activation during gastric stimulation (Study 1) was not significantly correlated with this network, activation in response to rectal stimulation showed a negative correlation (Study 2 and 3), and brain activation evoked by esophageal stimulation (Study 5) was positively correlated with this network.

**Supplementary Tables**

Table S1. Mean point-biserial correlations (p-values) between brain response to stimulation versus rest and resting-state networks for each study.

Table S2. Brain regions exhibiting increased activation during somatic stimulation versus rest.

Table S3. Brain regions exhibiting decreased activation during somatic stimulation versus rest.

Table S4. Brain regions exhibiting increased activation during visceral stimulation versus rest.

Table S5. Brain regions exhibiting decreased activation during visceral stimulation versus rest.

Table S6. Conjunction of brain regions exhibiting increased activation during visceral and somatic stimulation versus rest.

Table S7. Conjunction of brain regions exhibiting decreased activation during visceral and somatic stimulation versus rest.

Table S8. Mean point-biserial correlations (p-values) between brain response to stimulation versus rest and resting-state networks for each study.

Table S9. Brain regions exhibiting increased activity during stimulation versus rest (conjunction somatic – visceral) and greater amplitude of activation for somatic compared to visceral stimulation.

Table S10. Brain regions exhibiting increased activity during stimulation versus rest (conjunction somatic – visceral) and greater amplitude of activation for visceral compared to somatic stimulation.

Table S11. Brain regions exhibiting decreased activity during stimulation versus rest (conjunction somatic – visceral) and greater deactivation amplitude for somatic compared to visceral stimulation.

Table S12. Brain regions exhibiting decreased activity during stimulation versus rest (conjunction somatic – visceral) and greater deactivation amplitude for visceral compared to somatic stimulation.

## Supplementary Tables

Table S1. Mean spatial correlation (Cohen's *d*, *p*-value) between brain response to stimulation versus rest and brain signatures for each study.

| Study | NPS | PINES | Rejection | VPS |
|---|---|---|---|---|
| Study 1 (gastric pain) | *0.0387 (0.465, <.0001)* | 0.0080 (0.111, .1773) | 0.0082 (0.159, .0608) | -0.0120 (-0.171, .0451) |
| Study 2 (rectal discomfort 1) | *0.0462 (0.505, <.0001)* | -0.0029 (-0.036, .6470) | 0.0114 (0.120, .1485) | 0.0057 (0.074, .3587) |
| Study 3 (rectal discomfort 2) | *0.0512 (0.700, <.0001)* | -0.0003 (-0.006, .9361) | *0.0203 (0.332, .0002)* | *-0.0180 (-0.224, .0077)* |
| Study 4 (esophageal pain) | *0.0819 (1.563, <.0001)* | 0.0211 (0.403, .0122) | 0.0112 (0.164, .0640) | -0.0121 (-0.235, .0621) |
| Study 5 (vulvar pressure) | *0.0996 (0.688, <.0001)* | *0.0190 (0.224, <.0001)* | 0.0083 (0.157, .0440) | *-0.0121 (-0.158, .0052)* |
| Study 6 (thermal pain 1) | *0.1040 (1.165, <.0001)* | -0.0065 (-0.122, .1276) | *0.0345 (0.677, <.0001)* | *-0.0243 (-0.359, .0001)* |
| Study 7 (thermal pain 2) | *0.0706 (0.955, <.0001)* | 0.0086 (0.178, .2091) | *0.0138 (0.270, .0015)* | -0.0063 (-0.099, .2108) |

Note. P-values are reported in parenthesis and are based on a one-sample t-test of fisher-transformed correlation coefficients. *P*-values less than 0.0122 survive the false discovery rate threshold $q_{FDR} < .05$ (italic).

Table S2. Brain regions exhibiting increased activation during somatic stimulation versus rest.

| Cluster | Peak region | x | y | z | Volume (mm$^3$) | Peak t - statistic |
|---|---|---|---|---|---|---|
| Cluster 1 | Thalamus | 4 | -16 | -2 | 222008 | 14.50 |
| | Caudate | -20 | 20 | 14 | 816 | 6.31 |
| | Caudate | -16 | 4 | 24 | 352 | 3.75 |
| | Caudate | 18 | 0 | 24 | 384 | 3.72 |
| | Caudate | 18 | -16 | 22 | 352 | 3.12 |
| | Cerebellar Lingual | -6 | -48 | -20 | 8688 | 7.26 |
| | Cerebellum | -22 | -38 | -30 | 3920 | 6.46 |
| | Cerebellum | -2 | -82 | -32 | 3424 | 4.48 |
| | Claustrum | 38 | -6 | 0 | 5920 | 10.56 |
| | Culmen | -28 | -52 | -32 | 3144 | 5.74 |
| | Culmen | 8 | -38 | -22 | 4896 | 5.45 |
| | Culmen | -50 | -46 | -28 | 1888 | 4.76 |
| | Declive | -32 | -70 | -30 | 2888 | 5.99 |
| | Declive | -18 | -62 | -24 | 4408 | 5.66 |
| | Declive | -2 | -68 | -20 | 5416 | 5.16 |
| | Declive | 18 | -70 | -26 | 1952 | 4.26 |
| | Fusiform Gyrus | -44 | -28 | -18 | 608 | 2.98 |
| | Inferior Frontal Gyrus | 32 | 10 | -12 | 4424 | 7.57 |
| | Inferior Frontal Gyrus | 40 | 34 | 4 | 3816 | 6.57 |
| | Inferior Frontal Gyrus | -30 | 14 | -12 | 960 | 4.01 |
| | Inferior Parietal Lobule | 60 | -34 | 26 | 6608 | 9.70 |
| | Inferior Temporal Gyrus | -50 | -38 | -22 | 912 | 3.96 |
| | Inferior Temporal Gyrus | 54 | -26 | -30 | 368 | 2.89 |
| | Insula | 36 | 8 | 10 | 7928 | 14.50 |
| | Insula | 40 | -20 | 12 | 7048 | 12.15 |
| | Insula | 48 | 12 | 0 | 12880 | 11.91 |
| | Insula | 48 | -28 | 20 | 5952 | 10.49 |
| | Insula | 42 | -16 | -8 | 4960 | 10.19 |
| | Insula | -38 | 2 | 12 | 3744 | 9.98 |
| | Insula | -38 | 10 | 4 | 5392 | 9.98 |
| | Insula | -32 | 24 | 4 | 5232 | 9.41 |
| | Insula | 32 | 24 | 2 | 4288 | 9.41 |
| | Insula | -52 | -34 | 22 | 6920 | 8.26 |
| | Insula | -42 | 6 | -8 | 6432 | 7.53 |

| | | | | | | |
|---|---|---|---|---|---|---|
| | Insula | -42 | -10 | 0 | 4896 | 7.41 |
| | Insula | -40 | -20 | 14 | 6312 | 7.39 |
| | Insula | 48 | -28 | -12 | 2032 | 5.81 |
| | Insula | -40 | -22 | -4 | 952 | 4.70 |
| | Lentiform Nucleus | 30 | -12 | -2 | 1712 | 7.50 |
| | Lentiform Nucleus | 16 | -2 | -4 | 2872 | 6.54 |
| | Midbrain | 10 | -20 | -10 | 4912 | 9.25 |
| | Midbrain | 0 | -28 | -6 | 2496 | 6.19 |
| | Midbrain | -8 | -18 | -8 | 1488 | 4.52 |
| | Parahippocampal Gyrus | -36 | -42 | -2 | 304 | 2.98 |
| | Postcentral Gyrus | 60 | -18 | 20 | 6048 | 8.35 |
| | Postcentral Gyrus | -62 | -28 | 22 | 7648 | 7.55 |
| | Precentral Gyrus | 58 | 0 | 10 | 10944 | 11.67 |
| | Precentral Gyrus | -58 | 0 | 8 | 13464 | 11.19 |
| | Pyramis | -16 | -86 | -40 | 968 | 3.56 |
| | Pyramis | 10 | -86 | -34 | 856 | 2.85 |
| | Superior Temporal Gyrus | 44 | -32 | 0 | 480 | 4.99 |
| | Thalamus | 16 | -10 | 8 | 2168 | 6.04 |
| | Thalamus | 2 | -6 | 12 | 3656 | 5.55 |
| | Tuber | -44 | -60 | -32 | 4176 | 5.21 |
| | Tuber | -34 | -80 | -38 | 1984 | 3.67 |
| | Uvula | -16 | -74 | -34 | 4720 | 7.00 |
| Cluster 2 | Tuber | 50 | -60 | -36 | 1408 | 2.99 |
| | Cerebellar Tonsil | 54 | -54 | -42 | 240 | 2.67 |
| | Tuber | 46 | -60 | -34 | 688 | 2.99 |
| | Tuber | 54 | -60 | -38 | 480 | 2.95 |
| Cluster 3 | Tuber | 40 | -82 | -36 | 1088 | 3.14 |
| Cluster 4 | Lentiform Nucleus | -14 | -2 | -4 | 216 | 3.06 |
| Cluster 5 | Medial Frontal Gyrus | 10 | -12 | 56 | 86080 | 13.11 |
| | Anterior Cingulate | 0 | 20 | 30 | 6136 | 8.40 |
| | Anterior Cingulate | 0 | 24 | 18 | 864 | 5.59 |
| | Caudate | 10 | 22 | 8 | 144 | 3.12 |
| | Cingulate Gyrus | 6 | 4 | 44 | 7176 | 13.11 |
| | Cingulate Gyrus | -6 | 8 | 40 | 3672 | 12.14 |
| | Cingulate Gyrus | -6 | -6 | 48 | 5656 | 9.61 |
| | Cingulate Gyrus | 10 | -22 | 46 | 4008 | 7.10 |
| | Medial Frontal Gyrus | 6 | 2 | 60 | 9224 | 9.07 |

| | | | | | | |
|---|---|---|---|---|---|---|
| | Medial Frontal Gyrus | 4 | -10 | 68 | 14520 | 8.27 |
| | Medial Frontal Gyrus | 6 | -32 | 72 | 7992 | 5.66 |
| | Middle Frontal Gyrus | 50 | 0 | 46 | 2752 | 4.80 |
| | Postcentral Gyrus | 18 | -46 | 68 | 6648 | 12.42 |
| | Postcentral Gyrus | 8 | -50 | 76 | 1352 | 5.59 |
| | Precentral Gyrus | 28 | -30 | 68 | 8184 | 6.93 |
| | Precentral Gyrus | 18 | -28 | 62 | 6648 | 6.49 |
| | Precentral Gyrus | 36 | -18 | 50 | 1104 | 3.62 |
| Cluster 6 | * | -22 | -50 | 14 | 88 | 2.88 |
| | Posterior Cingulate | -20 | -48 | 12 | 40 | 2.88 |
| | Posterior Cingulate | -24 | -54 | 16 | 48 | 2.66 |
| Cluster 7 | Middle Frontal Gyrus | 32 | 44 | 26 | 2288 | 3.88 |
| Cluster 8 | Middle Frontal Gyrus | -34 | 40 | 28 | 3240 | 4.10 |
| Cluster 9 | Precentral Gyrus | -40 | -14 | 40 | 104 | 2.57 |
| Cluster 10 | Postcentral Gyrus | -18 | -40 | 62 | 8936 | 5.84 |
| | Cingulate Gyrus | -16 | -36 | 46 | 1424 | 4.35 |
| | Postcentral Gyrus | -20 | -34 | 62 | 3208 | 4.04 |
| | Superior Parietal Lobule | -20 | -46 | 66 | 4304 | 5.84 |

Note. **Average effect of somatic stimulation, independent of study.** The first entry for each cluster denotes the peak coordinate and volume of the entire cluster; subsequent entries for each cluster denote peak coordinates and volumes of sub-clusters. Peak statistics are reported at a threshold of $q_{FDR} < .05$. Coordinates are reported in MNI space. *No gray matter within 5mm of peak.

Table S3. Brain regions exhibiting decreased activation during somatic stimulation versus rest.

| Cluster | Peak region | x | y | z | Volume ($mm^3$) | Peak t - statistic |
|---|---|---|---|---|---|---|
| Cluster 1 | Superior Temporal Gyrus | 46 | 14 | -34 | 984 | -3.75 |
| | Superior Temporal Gyrus | 50 | 16 | -34 | 704 | -3.75 |
| | Superior Temporal Gyrus | 36 | 14 | -34 | 280 | -2.79 |
| Cluster 2 | Posterior Cingulate | -4 | -38 | 24 | 338208 | -10.42 |
| | Angular Gyrus | 48 | -66 | 34 | 4152 | -5.50 |
| | Anterior Cingulate | -4 | 56 | 0 | 5040 | -4.87 |
| | Anterior Cingulate | 6 | 48 | -2 | 4184 | -4.69 |
| | Anterior Cingulate | 6 | 38 | -8 | 2616 | -4.29 |
| | Anterior Cingulate | -8 | 36 | -8 | 2864 | -4.08 |
| | Cingulate Gyrus | -6 | -48 | 30 | 5904 | -10.42 |
| | Cingulate Gyrus | 4 | -38 | 36 | 6088 | -8.82 |
| | Cingulate Gyrus | 4 | -62 | 24 | 13240 | -8.40 |
| | Cuneus | -24 | -94 | -4 | 4368 | -4.32 |
| | Cuneus | 4 | -78 | 4 | 4696 | -3.95 |
| | Fusiform Gyrus | 32 | -36 | -20 | 4312 | -8.83 |
| | Fusiform Gyrus | -36 | -50 | -14 | 2424 | -6.87 |
| | Fusiform Gyrus | 40 | -58 | -12 | 5776 | -6.62 |
| | Inferior Occipital Gyrus | 42 | -78 | -8 | 5240 | -6.75 |
| | Inferior Occipital Gyrus | -34 | -84 | -12 | 4912 | -5.48 |
| | Inferior Occipital Gyrus | -12 | -90 | -10 | 2848 | -3.76 |
| | Inferior Parietal Lobule | -46 | -34 | 48 | 6176 | -6.56 |
| | Inferior Parietal Lobule | -48 | -60 | 42 | 8704 | -5.91 |
| | Inferior Parietal Lobule | -38 | -44 | 44 | 6504 | -5.36 |
| | Inferior Parietal Lobule | 38 | -44 | 44 | 4512 | -4.71 |
| | Inferior Parietal Lobule | 52 | -56 | 44 | 4056 | -4.49 |
| | Inferior Parietal Lobule | 50 | -34 | 50 | 2432 | -3.97 |
| | Inferior Temporal Gyrus | -44 | -76 | -4 | 4952 | -7.90 |
| | Inferior Temporal Gyrus | -42 | -64 | -10 | 3920 | -7.68 |
| | Inferior Temporal Gyrus | -50 | -54 | -12 | 2472 | -7.35 |
| | Lingual Gyrus | 24 | -82 | -10 | 6136 | -4.43 |
| | Medial Frontal Gyrus | -6 | 60 | 12 | 3424 | -5.21 |
| | Medial Frontal Gyrus | -10 | 46 | 36 | 2024 | -4.22 |
| | Medial Frontal Gyrus | 6 | 58 | 16 | 2912 | -3.88 |
| | Medial Frontal Gyrus | -8 | 56 | 24 | 2296 | -3.74 |

| | | | | | |
|---|---|---|---|---|---|
| Medial Frontal Gyrus | -6 | 46 | 18 | 2544 | -3.70 |
| Medial Frontal Gyrus | 2 | 26 | -10 | 1296 | -3.34 |
| Medial Frontal Gyrus | 8 | 10 | -24 | 272 | -3.31 |
| Medial Frontal Gyrus | -8 | 46 | -16 | 1472 | -3.28 |
| Medial Frontal Gyrus | 8 | 44 | 22 | 392 | -2.76 |
| Medial Frontal Gyrus | -4 | 62 | -16 | 488 | -2.69 |
| Middle Frontal Gyrus | -44 | 22 | 38 | 3752 | -6.47 |
| Middle Frontal Gyrus | -34 | 10 | 56 | 4184 | -5.97 |
| Middle Frontal Gyrus | -44 | 8 | 38 | 6352 | -5.47 |
| Middle Frontal Gyrus | 30 | 14 | 54 | 6696 | -5.34 |
| Middle Frontal Gyrus | -38 | 20 | 48 | 3312 | -4.86 |
| Middle Frontal Gyrus | -48 | 22 | 26 | 2776 | -4.12 |
| Middle Frontal Gyrus | 24 | 32 | 42 | 2280 | -4.06 |
| Middle Frontal Gyrus | -34 | -10 | 64 | 2504 | -3.84 |
| Middle Frontal Gyrus | -16 | 42 | -20 | 504 | -3.11 |
| Middle Occipital Gyrus | -32 | -88 | 14 | 6616 | -5.48 |
| Middle Occipital Gyrus | 24 | -96 | 0 | 3704 | -5.48 |
| Middle Occipital Gyrus | 38 | -86 | 8 | 5528 | -5.10 |
| Middle Temporal Gyrus | -48 | -60 | 24 | 6216 | -5.97 |
| Middle Temporal Gyrus | 44 | -78 | 26 | 3232 | -5.69 |
| Middle Temporal Gyrus | 54 | -52 | -10 | 1328 | -4.76 |
| Middle Temporal Gyrus | -58 | -22 | -12 | 2952 | -4.62 |
| Middle Temporal Gyrus | -56 | -4 | -20 | 2320 | -4.38 |
| Middle Temporal Gyrus | -60 | -38 | -4 | 4504 | -4.07 |
| Middle Temporal Gyrus | -64 | -8 | -16 | 504 | -3.21 |
| Middle Temporal Gyrus | -54 | 2 | -32 | 384 | -2.96 |
| Parahippocampal Gyrus | -28 | -36 | -16 | 2616 | -8.00 |
| Parahippocampal Gyrus | 26 | -48 | -14 | 6048 | -6.70 |
| Parahippocampal Gyrus | 18 | -38 | 2 | 4888 | -5.92 |
| Parahippocampal Gyrus | 26 | -14 | -20 | 1784 | -4.00 |
| Parahippocampal Gyrus | -24 | -14 | -20 | 2448 | -3.90 |
| Parahippocampal Gyrus | 18 | -6 | -22 | 232 | -3.06 |
| Postcentral Gyrus | -52 | -22 | 54 | 1752 | -4.96 |
| Postcentral Gyrus | -42 | -28 | 62 | 3064 | -4.73 |
| Posterior Cingulate | -4 | -54 | 10 | 8320 | -7.09 |
| Precentral Gyrus | 36 | 22 | 42 | 6152 | -4.60 |
| Precentral Gyrus | -34 | -20 | 68 | 1976 | -4.16 |

| | | | | | | |
|---|---|---|---|---|---|---|
| | Precuneus | 4 | -54 | 40 | 8224 | -9.81 |
| | Precuneus | 20 | -62 | 50 | 6864 | -6.11 |
| | Precuneus | 24 | -76 | 50 | 2968 | -6.09 |
| | Precuneus | 34 | -76 | 32 | 8184 | -6.06 |
| | Precuneus | -28 | -78 | 34 | 5928 | -5.36 |
| | Precuneus | -36 | -70 | 32 | 4216 | -4.45 |
| | Rectal Gyrus | -10 | 24 | -22 | 1136 | -3.49 |
| | Subcallosal Gyrus | -4 | 14 | -14 | 576 | -2.84 |
| | Superior Frontal Gyrus | -16 | 38 | 48 | 4448 | -5.73 |
| | Superior Frontal Gyrus | -22 | 24 | 54 | 5904 | -5.50 |
| | Superior Frontal Gyrus | -18 | 56 | 32 | 2048 | -4.06 |
| | Superior Frontal Gyrus | 14 | 54 | 36 | 1064 | -4.02 |
| | Superior Frontal Gyrus | 22 | 34 | 52 | 1800 | -3.86 |
| | Superior Frontal Gyrus | 16 | 60 | 18 | 856 | -3.24 |
| | Superior Parietal Lobule | -32 | -60 | 52 | 8288 | -8.92 |
| | Superior Parietal Lobule | 34 | -64 | 52 | 7456 | -7.13 |
| | Superior Parietal Lobule | -26 | -72 | 52 | 4608 | -6.32 |
| | Superior Temporal Gyrus | 56 | -62 | 22 | 2840 | -5.46 |
| | Superior Temporal Gyrus | 46 | -56 | 22 | 3752 | -5.33 |
| | Thalamus | -16 | -30 | 2 | 2272 | -4.18 |
| | Uncus | -26 | -6 | -26 | 200 | -2.79 |
| Cluster 3 | Middle Temporal Gyrus | -48 | 10 | -30 | 824 | -3.23 |
| | Middle Temporal Gyrus | -56 | 12 | -28 | 416 | -3.23 |
| | Superior Temporal Gyrus | -42 | 10 | -32 | 408 | -3.04 |
| Cluster 4 | Middle Temporal Gyrus | 62 | -12 | -14 | 3032 | -4.45 |
| | Middle Temporal Gyrus | 60 | -12 | -14 | 2584 | -4.45 |
| | Middle Temporal Gyrus | 70 | -14 | -10 | 448 | -3.51 |
| Cluster 5 | Superior Frontal Gyrus | 38 | 46 | -18 | 144 | -3.05 |
| Cluster 6 | Middle Frontal Gyrus | -34 | 50 | -10 | 448 | -2.73 |
| | Middle Frontal Gyrus | -32 | 52 | -8 | 296 | -2.73 |
| | Superior Frontal Gyrus | -36 | 50 | -14 | 152 | -2.62 |
| Cluster 7 | Inferior Frontal Gyrus | -50 | 32 | -8 | 1288 | -3.38 |
| Cluster 8 | Superior Frontal Gyrus | -26 | 58 | 8 | 352 | -2.88 |

Note. **Average effect of somatic stimulation, independent of study.** The first entry for each cluster denotes the peak coordinate and volume of the entire cluster; subsequent entries for each cluster denote peak coordinates and volumes of sub-clusters. Peak statistics are reported at a threshold of $q_{FDR} < .05$. Coordinates are reported in MNI space.

Table S4. Brain regions exhibiting increased activation during visceral stimulation versus rest.

| Cluster | Peak region | x | y | z | Volume ($mm^3$) | Peak t - statistic |
|---|---|---|---|---|---|---|
| Cluster 1 | Thalamus | 6 | -18 | 10 | 523200 | 13.99 |
| | * | 6 | -40 | -48 | 336 | 3.07 |
| | Cerebellar Tonsil | 32 | -58 | -48 | 992 | 3.27 |
| | Cerebellum | 18 | -44 | -36 | 1936 | 4.25 |
| | Cerebellum | -12 | -42 | -36 | 3048 | 3.47 |
| | Cingulate Gyrus | 0 | -24 | 26 | 4256 | 6.19 |
| | Cingulate Gyrus | 4 | 20 | 34 | 15016 | 9.58 |
| | Cingulate Gyrus | -10 | -24 | 40 | 3280 | 5.48 |
| | Cingulate Gyrus | 10 | -24 | 40 | 4224 | 5.73 |
| | Cingulate Gyrus | -6 | 10 | 40 | 11088 | 10.97 |
| | Claustrum | 40 | -8 | -6 | 7976 | 9.41 |
| | Claustrum | -38 | 10 | 2 | 20248 | 12.16 |
| | Culmen | 36 | -52 | -32 | 7080 | 6.42 |
| | Culmen | -32 | -54 | -34 | 7360 | 6.79 |
| | Culmen | -2 | -56 | -26 | 10392 | 5.79 |
| | Culmen | 10 | -64 | -16 | 11752 | 6.92 |
| | Cuneus | -10 | -72 | 8 | 8952 | 6.17 |
| | Declive | -42 | -62 | -30 | 2856 | 5.48 |
| | Declive | -30 | -76 | -24 | 4264 | 6.07 |
| | Declive | 24 | -62 | -26 | 11048 | 7.82 |
| | Declive | -20 | -64 | -22 | 15240 | 8.91 |
| | Hippocampus | 22 | -26 | -6 | 3640 | 6.10 |
| | Inferior Frontal Gyrus | 42 | 42 | 12 | 7528 | 5.86 |
| | Inferior Frontal Gyrus | -54 | 4 | 30 | 5792 | 5.52 |
| | Inferior Frontal Gyrus | 54 | 10 | 32 | 8576 | 6.97 |
| | Inferior Occipital Gyrus | 14 | -90 | -10 | 872 | 3.64 |
| | Inferior Parietal Lobule | 60 | -30 | 36 | 6360 | 8.76 |
| | Inferior Parietal Lobule | 36 | -50 | 42 | 5440 | 6.16 |
| | Inferior Parietal Lobule | -36 | -48 | 42 | 6440 | 5.50 |
| | Inferior Parietal Lobule | 50 | -40 | 44 | 5664 | 5.32 |
| | Inferior Parietal Lobule | -54 | -40 | 46 | 6440 | 6.65 |
| | Inferior Parietal Lobule | -32 | -44 | 60 | 696 | 3.06 |
| | Inferior Semi-Lunar Lobule | 16 | -72 | -46 | 5136 | 6.16 |
| | Inferior Semi-Lunar Lobule | -22 | -70 | -46 | 7008 | 5.93 |

| | | | | | |
|---|---|---|---|---|---|
| Insula | 42 | 12 | -4 | 11792 | 10.85 |
| Insula | 36 | 22 | 4 | 10656 | 9.94 |
| Insula | -38 | -16 | 8 | 7416 | 8.66 |
| Insula | -50 | -36 | 20 | 6168 | 7.85 |
| Insula | 50 | -32 | 20 | 7776 | 11.73 |
| Lentiform Nucleus | 20 | 2 | -6 | 8384 | 7.78 |
| Lentiform Nucleus | -30 | -10 | -6 | 4256 | 8.92 |
| Lentiform Nucleus | -16 | 4 | 0 | 6216 | 6.30 |
| Lentiform Nucleus | 28 | -10 | 8 | 5040 | 8.10 |
| Lingual Gyrus | -8 | -84 | -6 | 10168 | 5.04 |
| Lingual Gyrus | 12 | -82 | -4 | 5208 | 5.12 |
| Medial Frontal Gyrus | -18 | 2 | 52 | 3432 | 8.00 |
| Medial Frontal Gyrus | 8 | 4 | 56 | 12136 | 9.45 |
| Medial Frontal Gyrus | -4 | -6 | 60 | 5848 | 7.61 |
| Medial Frontal Gyrus | -16 | 2 | 66 | 1624 | 5.27 |
| Midbrain | 10 | -16 | -14 | 4032 | 6.98 |
| Midbrain | -6 | -20 | -10 | 7776 | 6.82 |
| Middle Frontal Gyrus | 46 | 36 | -8 | 1392 | 3.65 |
| Middle Frontal Gyrus | 32 | 40 | 24 | 6232 | 6.06 |
| Middle Frontal Gyrus | -36 | 42 | 20 | 13144 | 7.95 |
| Middle Frontal Gyrus | 40 | 42 | 32 | 2760 | 5.05 |
| Middle Frontal Gyrus | -40 | 32 | 36 | 1360 | 4.69 |
| Middle Frontal Gyrus | -38 | -6 | 50 | 6272 | 6.05 |
| Middle Frontal Gyrus | -18 | 8 | 64 | 104 | 2.85 |
| Middle Temporal Gyrus | 58 | -46 | 4 | 7584 | 5.72 |
| Paracentral Lobule | -22 | -44 | 54 | 2152 | 3.65 |
| Parahippocampal Gyrus | -20 | -26 | -8 | 2824 | 6.23 |
| Postcentral Gyrus | -60 | -24 | 22 | 15664 | 12.13 |
| Postcentral Gyrus | 62 | -22 | 20 | 11616 | 12.39 |
| Postcentral Gyrus | -46 | -24 | 40 | 7304 | 6.79 |
| Postcentral Gyrus | -22 | -32 | 58 | 1456 | 4.46 |
| Posterior Cingulate | 12 | -68 | 6 | 9144 | 5.34 |
| Precentral Gyrus | 48 | 0 | 10 | 18192 | 12.80 |
| Precentral Gyrus | -48 | -8 | 26 | 4856 | 7.73 |
| Precentral Gyrus | 50 | -12 | 30 | 4680 | 6.67 |
| Precentral Gyrus | 44 | 2 | 40 | 11208 | 8.29 |
| Precuneus | -10 | -72 | 36 | 6352 | 5.88 |

| | | | | | | |
|---|---|---|---|---|---|---|
| | Precuneus | 12 | -70 | 34 | 7200 | 6.93 |
| | Pyramis | -4 | -76 | -34 | 7224 | 5.34 |
| | Subcallosal Gyrus | -28 | 4 | -12 | 6680 | 8.54 |
| | Superior Temporal Gyrus | -56 | -54 | 4 | 8112 | 6.36 |
| | Superior Temporal Gyrus | -54 | -2 | 6 | 10840 | 13.99 |
| | Thalamus | 6 | -18 | -2 | 3704 | 7.92 |
| | Thalamus | -14 | -16 | 6 | 2864 | 8.53 |
| | Thalamus | 12 | -10 | 6 | 3416 | 8.62 |
| Cluster 2 | Inferior Temporal Gyrus | -48 | -16 | -38 | 80 | 3.37 |
| Cluster 3 | Middle Frontal Gyrus | 26 | 54 | -14 | 120 | 2.80 |
| Cluster 4 | Postcentral Gyrus | 22 | -30 | 56 | 384 | 3.57 |

Note. **Average effect of visceral stimulation, independent of study.** The first entry for each cluster denotes the peak coordinate and volume of the entire cluster; subsequent entries for each cluster denote peak coordinates and volumes of sub-clusters. Peak statistics are reported at a threshold of $q_{FDR} < .05$. Coordinates are reported in MNI space. *No gray matter within 5mm of peak.

Table S5. Brain regions exhibiting decreased activation during visceral stimulation versus rest.

| Cluster | Peak region | x | y | z | Volume (mm$^3$) | Peak t – statistic |
|---|---|---|---|---|---|---|
| Cluster 1 | Culmen | 38 | -36 | -38 | 1488 | -4.32 |
| | Cerebellar Tonsil | 24 | -38 | -48 | 176 | -3.01 |
| | Cerebellar Tonsil | 46 | -44 | -42 | 248 | -3.28 |
| | Cerebellar Tonsil | 36 | -36 | -42 | 384 | -3.97 |
| | Culmen | 46 | -40 | -32 | 208 | -4.32 |
| | Parahippocampal Gyrus | 36 | -24 | -32 | 144 | -2.90 |
| | Parahippocampal Gyrus | 40 | -32 | -28 | 328 | -3.68 |
| Cluster 2 | Superior Temporal Gyrus | 40 | 6 | -34 | 8912 | -5.63 |
| | Inferior Temporal Gyrus | 40 | 0 | -48 | 296 | -5.05 |
| | Inferior Temporal Gyrus | 60 | -8 | -16 | 1016 | -3.17 |
| | Middle Temporal Gyrus | 44 | 8 | -38 | 2288 | -5.63 |
| | Middle Temporal Gyrus | 58 | 2 | -28 | 880 | -4.70 |
| | Superior Temporal Gyrus | 42 | 20 | -38 | 1096 | -4.37 |
| | Superior Temporal Gyrus | 28 | 12 | -38 | 1160 | -3.94 |
| | Superior Temporal Gyrus | 40 | 12 | -28 | 296 | -2.99 |
| | Uncus | 24 | 0 | -34 | 976 | -4.53 |
| | Uncus | 14 | -6 | -30 | 904 | -4.51 |
| Cluster 3 | Superior Temporal Gyrus | -38 | 6 | -34 | 9064 | -4.17 |
| | Brainstem | -8 | -12 | -34 | 120 | -3.03 |
| | Middle Temporal Gyrus | -46 | 0 | -42 | 904 | -3.83 |
| | Middle Temporal Gyrus | -38 | 8 | -36 | 1536 | -3.99 |
| | Middle Temporal Gyrus | -52 | 0 | -32 | 720 | -3.49 |
| | Middle Temporal Gyrus | -58 | 6 | -24 | 664 | -3.50 |
| | Parahippocampal Gyrus | -20 | -24 | -32 | 88 | -3.29 |
| | Parahippocampal Gyrus | -14 | -14 | -32 | 584 | -4.17 |
| | Superior Temporal Gyrus | -30 | 6 | -42 | 280 | -3.47 |
| | Superior Temporal Gyrus | -32 | 16 | -36 | 1144 | -3.78 |
| | Superior Temporal Gyrus | -48 | 16 | -34 | 1048 | -3.63 |
| | Superior Temporal Gyrus | -44 | 18 | -26 | 656 | -3.19 |
| | Uncus | -22 | -6 | -34 | 320 | -3.56 |
| | Uncus | -24 | 4 | -32 | 912 | -3.71 |
| | Uncus | -16 | 4 | -26 | 88 | -2.57 |
| Cluster 4 | Cerebellar Tonsil | -28 | -34 | -42 | 408 | -3.60 |

| | | | | | | |
|---|---|---|---|---|---|---|
| Cluster 5 | Brainstem | 14 | -24 | -36 | 104 | -2.84 |
| Cluster 6 | Fusiform Gyrus | -44 | -32 | -28 | 168 | -3.37 |
| Cluster 7 | Inferior Temporal Gyrus | 58 | -26 | -26 | 88 | -2.89 |
| Cluster 8 | Medial Frontal Gyrus | -4 | 50 | 20 | 64072 | -7.27 |
| | Anterior Cingulate | 0 | 22 | -2 | 1360 | -4.97 |
| | Medial Frontal Gyrus | 14 | 16 | -22 | 224 | -4.21 |
| | Medial Frontal Gyrus | 0 | 14 | -18 | 2320 | -7.11 |
| | Medial Frontal Gyrus | -2 | 64 | -10 | 528 | -3.65 |
| | Medial Frontal Gyrus | -4 | 48 | -12 | 4472 | -6.57 |
| | Medial Frontal Gyrus | 2 | 34 | -10 | 5088 | -5.55 |
| | Medial Frontal Gyrus | -10 | 38 | -4 | 2208 | -4.98 |
| | Medial Frontal Gyrus | 0 | 56 | 0 | 6880 | -4.98 |
| | Medial Frontal Gyrus | 14 | 38 | -2 | 488 | -4.04 |
| | Medial Frontal Gyrus | -16 | 64 | 10 | 1728 | -4.74 |
| | Medial Frontal Gyrus | 8 | 62 | 16 | 3272 | -4.85 |
| | Medial Frontal Gyrus | -8 | 60 | 18 | 5976 | -7.27 |
| | Medial Frontal Gyrus | -6 | 48 | 42 | 3224 | -4.81 |
| | Middle Frontal Gyrus | 26 | 28 | 46 | 6384 | -5.67 |
| | Middle Frontal Gyrus | -28 | 20 | 48 | 5864 | -6.08 |
| | Orbital Gyrus | -6 | 48 | -20 | 384 | -3.48 |
| | Superior Frontal Gyrus | -12 | 54 | 32 | 2664 | -5.16 |
| | Superior Frontal Gyrus | 6 | 54 | 36 | 2200 | -3.72 |
| | Superior Frontal Gyrus | -18 | 36 | 46 | 6584 | -5.35 |
| | Superior Frontal Gyrus | 20 | 44 | 44 | 848 | -3.87 |
| | Superior Frontal Gyrus | 22 | 32 | 54 | 1376 | -4.43 |
| Cluster 9 | Inferior Frontal Gyrus | 16 | 36 | -24 | 120 | -2.77 |
| Cluster 10 | Parahippocampal Gyrus | 30 | -16 | -22 | 640 | -3.17 |
| Cluster 11 | Parahippocampal Gyrus | -28 | -16 | -22 | 544 | -3.26 |
| Cluster 12 | Posterior Cingulate | -4 | -52 | 14 | 22240 | -4.89 |
| | * | -28 | -54 | 10 | 328 | -3.24 |
| | Caudate | 30 | -46 | 12 | 552 | -4.48 |
| | Caudate | 24 | -36 | 10 | 552 | -4.51 |
| | Caudate | 22 | -28 | 24 | 232 | -3.37 |
| | Cingulate Gyrus | -16 | -52 | 24 | 880 | -3.48 |
| | Cingulate Gyrus | 0 | -54 | 28 | 8280 | -4.62 |
| | Parahippocampal Gyrus | 28 | -30 | -20 | 96 | -2.65 |
| | Parahippocampal Gyrus | 38 | -32 | -16 | 408 | -2.93 |

| | | | | | | |
|---|---|---|---|---|---|---|
| | Parahippocampal Gyrus | -32 | -40 | -10 | 2000 | -4.31 |
| | Parahippocampal Gyrus | 34 | -42 | -4 | 1184 | -4.39 |
| | Parahippocampal Gyrus | -32 | -48 | -2 | 368 | -4.12 |
| | Posterior Cingulate | -14 | -52 | 12 | 2408 | -4.89 |
| | Posterior Cingulate | 14 | -50 | 14 | 1608 | -4.32 |
| | Sub-Gyral | -24 | -44 | 4 | 1088 | -3.67 |
| | Thalamus | 18 | -36 | 8 | 776 | -4.30 |
| | Thalamus | -14 | -38 | 8 | 1480 | -4.18 |
| Cluster 13 | Middle Temporal Gyrus | -62 | -14 | -14 | 2472 | -3.63 |
| | Middle Temporal Gyrus | -60 | -18 | -18 | 432 | -3.24 |
| | Middle Temporal Gyrus | -60 | -10 | -14 | 1240 | -3.48 |
| | Middle Temporal Gyrus | -66 | -18 | -8 | 800 | -3.63 |
| Cluster 14 | Precuneus | 2 | -64 | 62 | 46024 | -7.80 |
| | Cuneus | -26 | -92 | 26 | 272 | -3.52 |
| | Cuneus | 8 | -92 | 30 | 368 | -3.93 |
| | Cuneus | -12 | -92 | 32 | 144 | -3.98 |
| | Cuneus | 20 | -90 | 32 | 888 | -5.31 |
| | Cuneus | -20 | -88 | 36 | 792 | -5.21 |
| | Medial Frontal Gyrus | 2 | -30 | 76 | 2496 | -5.68 |
| | Medial Frontal Gyrus | 8 | -22 | 78 | 1584 | -6.02 |
| | Middle Frontal Gyrus | 34 | -12 | 68 | 912 | -4.29 |
| | Middle Temporal Gyrus | -46 | -68 | 26 | 8656 | -7.80 |
| | Postcentral Gyrus | -8 | -62 | 70 | 464 | -3.82 |
| | Postcentral Gyrus | -4 | -44 | 74 | 624 | -3.62 |
| | Postcentral Gyrus | 6 | -42 | 78 | 576 | -4.18 |
| | Precentral Gyrus | 38 | -22 | 52 | 3712 | -5.31 |
| | Precentral Gyrus | 48 | -18 | 56 | 1056 | -4.93 |
| | Precentral Gyrus | 38 | -30 | 66 | 1000 | -5.26 |
| | Precentral Gyrus | 42 | -22 | 64 | 1144 | -5.31 |
| | Precentral Gyrus | 26 | -22 | 74 | 936 | -5.09 |
| | Precuneus | 44 | -72 | 34 | 3032 | -5.17 |
| | Precuneus | -34 | -78 | 38 | 3096 | -5.62 |
| | Precuneus | 6 | -86 | 44 | 440 | -5.13 |
| | Precuneus | 26 | -82 | 44 | 896 | -5.60 |
| | Precuneus | -20 | -82 | 44 | 472 | -4.81 |
| | Precuneus | 12 | -78 | 54 | 424 | -4.88 |
| | Precuneus | -22 | -78 | 50 | 352 | -4.58 |

| | | | | | | |
|---|---|---|---|---|---|---|
| | Precuneus | 2 | -62 | 66 | 360 | -3.94 |
| | Precuneus | 10 | -68 | 66 | 864 | -4.75 |
| | Superior Frontal Gyrus | 18 | -12 | 76 | 928 | -4.38 |
| | Superior Occipital Gyrus | 34 | -84 | 30 | 920 | -4.36 |
| | Superior Parietal Lobule | -12 | -72 | 62 | 344 | -4.32 |
| | Superior Parietal Lobule | -20 | -66 | 66 | 352 | -4.15 |
| | Superior Parietal Lobule | 30 | -56 | 68 | 552 | -3.77 |
| | Superior Parietal Lobule | 20 | -64 | 68 | 920 | -4.21 |
| | Superior Temporal Gyrus | 44 | -58 | 20 | 1616 | -4.21 |
| | Superior Temporal Gyrus | 54 | -62 | 24 | 4832 | -5.07 |
| Cluster 15 | Caudate | -20 | -22 | 26 | 368 | -3.76 |
| | Caudate | -24 | -30 | 26 | 152 | -3.40 |
| | Caudate | -20 | -18 | 26 | 216 | -3.76 |
| Cluster 16 | Precentral Gyrus | -56 | -6 | 52 | 112 | -3.99 |
| Cluster 17 | Medial Frontal Gyrus | 2 | -26 | 60 | 2736 | -5.17 |
| Cluster 18 | Precentral Gyrus | -40 | -16 | 68 | 1480 | -6.16 |
| | Precentral Gyrus | -50 | -16 | 60 | 208 | -4.02 |
| | Precentral Gyrus | -46 | -10 | 62 | 184 | -5.06 |
| | Precentral Gyrus | -40 | -14 | 66 | 512 | -6.16 |
| | Precentral Gyrus | -38 | -24 | 70 | 224 | -5.80 |
| | Precentral Gyrus | -30 | -20 | 76 | 200 | -4.09 |
| | Superior Frontal Gyrus | -24 | -6 | 74 | 152 | -4.00 |
| Cluster 19 | Postcentral Gyrus | -38 | -34 | 70 | 432 | -4.39 |
| | Postcentral Gyrus | -48 | -28 | 64 | 104 | -4.39 |
| | Postcentral Gyrus | -42 | -34 | 68 | 136 | -3.66 |
| | Postcentral Gyrus | -32 | -38 | 74 | 192 | -3.56 |

Note. **Average effect of visceral stimulation, independent of study.** The first entry for each cluster denotes the peak coordinate and volume of the entire cluster; subsequent entries for each cluster denote peak coordinates and volumes of sub-clusters. Peak statistics are reported at a threshold of $q_{FDR} < .05$. Coordinates are reported in MNI space. *No gray matter within 5mm of peak.

Table S6. Conjunction of brain regions exhibiting increased activation during visceral and somatic stimulation versus rest.

| Cluster | Peak region | x | y | z | Volume ($mm^3$) |
|---|---|---|---|---|---|
| Cluster 1 | Lentiform Nucleus | 28 | -18 | -2 | 122528 |
| | Cerebellum | -26 | -44 | -32 | 1312 |
| | Cerebellum | -12 | -44 | -30 | 544 |
| | Claustrum | 34 | 0 | -6 | 2416 |
| | Claustrum | 28 | 18 | 2 | 1176 |
| | Claustrum | 36 | -24 | 8 | 920 |
| | Claustrum | 30 | 24 | 8 | 944 |
| | Culmen | -34 | -54 | -32 | 2216 |
| | Culmen | 12 | -44 | -28 | 376 |
| | Culmen | 2 | -50 | -26 | 952 |
| | Culmen | 4 | -32 | -22 | 728 |
| | Culmen | -14 | -46 | -24 | 1576 |
| | Culmen | -8 | -56 | -22 | 2640 |
| | Culmen | -4 | -60 | -14 | 2080 |
| | Culmen | 2 | -50 | -16 | 904 |
| | Declive | -44 | -56 | -30 | 1072 |
| | Declive | 16 | -74 | -28 | 568 |
| | Declive | 26 | -72 | -28 | 176 |
| | Declive | -32 | -68 | -28 | 2320 |
| | Declive | -20 | -74 | -28 | 1544 |
| | Declive | 22 | -66 | -26 | 456 |
| | Declive | 14 | -68 | -24 | 728 |
| | Declive | -22 | -60 | -22 | 2160 |
| | Declive | -10 | -68 | -22 | 1768 |
| | Declive of Vermis | 2 | -76 | -28 | 1744 |
| | Declive of Vermis | -2 | -72 | -16 | 992 |
| | Dentate | -16 | -58 | -30 | 1192 |
| | Extra-Nuclear | 34 | 18 | -10 | 1064 |
| | Extra-Nuclear | 38 | 22 | 0 | 1624 |
| | Hypothalamus | 8 | -6 | -8 | 600 |
| | Inferior Frontal Gyrus | 28 | 26 | -4 | 632 |
| | Inferior Frontal Gyrus | 38 | 28 | -6 | 1080 |
| | Inferior Frontal Gyrus | 48 | 22 | -2 | 1920 |
| | Inferior Frontal Gyrus | 40 | 40 | 6 | 1824 |

| | | | | |
|---|---|---|---|---|
| Inferior Frontal Gyrus | 42 | 24 | 10 | 2456 |
| Inferior Parietal Lobule | 64 | -38 | 26 | 672 |
| Inferior Parietal Lobule | 60 | -28 | 26 | 2784 |
| Inferior Parietal Lobule | 48 | -36 | 26 | 664 |
| Inferior Parietal Lobule | 64 | -30 | 34 | 1072 |
| Inferior Parietal Lobule | 64 | -40 | 36 | 568 |
| Insula | 44 | -16 | -4 | 1392 |
| Insula | 46 | 8 | -4 | 3288 |
| Insula | 38 | 12 | 6 | 2680 |
| Insula | 40 | -18 | 8 | 2056 |
| Insula | 48 | -12 | 8 | 3272 |
| Insula | 36 | -10 | 14 | 2016 |
| Insula | 42 | -26 | 18 | 2128 |
| Insula | 52 | -38 | 20 | 1472 |
| Insula | 42 | 12 | 18 | 896 |
| Lentiform Nucleus | 24 | -6 | -6 | 960 |
| Lentiform Nucleus | 28 | 10 | -6 | 872 |
| Lentiform Nucleus | 32 | -16 | -2 | 1912 |
| Lentiform Nucleus | 16 | 2 | 2 | 1896 |
| Lentiform Nucleus | 26 | -8 | 6 | 1248 |
| Lentiform Nucleus | 30 | 4 | 10 | 2064 |
| Parahippocampal Gyrus | 28 | 6 | -18 | 504 |
| Postcentral Gyrus | 60 | -18 | 22 | 2928 |
| Postcentral Gyrus | 50 | -26 | 30 | 1384 |
| Precentral Gyrus | 58 | 12 | 10 | 2320 |
| Precentral Gyrus | 64 | -6 | 12 | 1120 |
| Precentral Gyrus | 46 | 4 | 12 | 3048 |
| Precentral Gyrus | 54 | 0 | 16 | 3176 |
| Precentral Gyrus | 62 | 4 | 20 | 1240 |
| Pyramis | -8 | -70 | -34 | 1904 |
| Red Nucleus | 2 | -28 | -12 | 2280 |
| Red Nucleus | -2 | -18 | -6 | 1984 |
| Sub-Gyral | 40 | -10 | -10 | 1536 |
| Substania Nigra | 10 | -22 | -14 | 1344 |
| Substania Nigra | -8 | -20 | -12 | 656 |
| Substania Nigra | 14 | -26 | -8 | 1272 |
| Subthalamic Nucleus | 16 | -12 | -10 | 648 |

| | | | | | |
|---|---|---|---|---|---|
| | Superior Temporal Gyrus | 42 | -2 | -18 | 504 |
| | Superior Temporal Gyrus | 38 | 6 | -16 | 784 |
| | Superior Temporal Gyrus | 48 | 16 | -12 | 992 |
| | Superior Temporal Gyrus | 48 | -26 | -10 | 304 |
| | Superior Temporal Gyrus | 54 | 4 | -4 | 1304 |
| | Superior Temporal Gyrus | 60 | 4 | 2 | 1136 |
| | Superior Temporal Gyrus | 60 | -6 | 4 | 1104 |
| | Superior Temporal Gyrus | 62 | -34 | 16 | 864 |
| | Supramarginal Gyrus | 56 | -38 | 34 | 824 |
| | Thalamus | 10 | -12 | 0 | 1712 |
| | Thalamus | 16 | -16 | 10 | 432 |
| | Transverse Temporal Gyrus | 50 | -26 | 12 | 1664 |
| | Transverse Temporal Gyrus | 62 | -20 | 12 | 1376 |
| | Tuber | -42 | -66 | -34 | 832 |
| | Uvula | -14 | -78 | -36 | 1176 |
| | Uvula | -32 | -72 | -36 | 400 |
| | Uvula | -24 | -68 | -36 | 696 |
| | Uvula of Vermis | 0 | -74 | -42 | 464 |
| Cluster 2 | Tuber | 44 | -60 | -34 | 488 |
| Cluster 3 | Precentral Gyrus | -48 | -6 | 8 | 57376 |
| | Claustrum | -38 | -16 | 0 | 1184 |
| | Claustrum | -36 | -6 | 8 | 2128 |
| | Extra-Nuclear | -36 | 24 | -4 | 1632 |
| | Inferior Frontal Gyrus | -34 | 10 | -12 | 1224 |
| | Inferior Frontal Gyrus | -46 | 14 | -2 | 2360 |
| | Inferior Parietal Lobule | -60 | -42 | 28 | 2176 |
| | Inferior Parietal Lobule | -60 | -30 | 28 | 2888 |
| | Insula | -40 | -18 | -8 | 608 |
| | Insula | -28 | 20 | -6 | 944 |
| | Insula | -38 | 6 | -2 | 2600 |
| | Insula | -36 | -24 | 8 | 1400 |
| | Insula | -32 | 22 | 6 | 3616 |
| | Insula | -48 | -8 | 16 | 1872 |
| | Insula | -34 | 8 | 12 | 2560 |
| | Insula | -40 | -20 | 16 | 2144 |
| | Insula | -48 | -30 | 22 | 2664 |
| | Parahippocampal Gyrus | -28 | 6 | -16 | 232 |

| | | | | | |
|---|---|---|---|---|---|
| | Postcentral Gyrus | -66 | -22 | 20 | 1256 |
| | Postcentral Gyrus | -56 | -18 | 24 | 1104 |
| | Precentral Gyrus | -54 | -2 | 8 | 3200 |
| | Precentral Gyrus | -62 | -2 | 16 | 1640 |
| | Precentral Gyrus | -52 | 4 | 16 | 1920 |
| | Sub-Gyral | -40 | -6 | -16 | 400 |
| | Sub-Gyral | -40 | -4 | -10 | 1528 |
| | Superior Temporal Gyrus | -52 | 12 | -10 | 808 |
| | Superior Temporal Gyrus | -48 | 0 | -4 | 1520 |
| | Superior Temporal Gyrus | -60 | -4 | 0 | 1448 |
| | Superior Temporal Gyrus | -54 | 10 | 4 | 2336 |
| | Superior Temporal Gyrus | -48 | -16 | 6 | 1768 |
| | Superior Temporal Gyrus | -50 | -34 | 14 | 1488 |
| | Superior Temporal Gyrus | -62 | -34 | 18 | 1592 |
| | Transverse Temporal Gyrus | -62 | -14 | 8 | 1176 |
| | Transverse Temporal Gyrus | -58 | -20 | 16 | 1960 |
| Cluster 4 | Lentiform Nucleus | -14 | -2 | -4 | 216 |
| Cluster 5 | Middle Frontal Gyrus | 32 | 44 | 26 | 2264 |
| | Middle Frontal Gyrus | 34 | 44 | 26 | 1376 |
| | Superior Frontal Gyrus | 28 | 42 | 22 | 888 |
| Cluster 6 | Cingulate Gyrus | 2 | 4 | 46 | 35464 |
| | Anterior Cingulate | -4 | 26 | 22 | 624 |
| | Anterior Cingulate | 4 | 20 | 24 | 1344 |
| | Cingulate Gyrus | -8 | 20 | 30 | 1040 |
| | Cingulate Gyrus | 6 | 10 | 32 | 1672 |
| | Cingulate Gyrus | -6 | 10 | 34 | 1384 |
| | Cingulate Gyrus | 2 | 22 | 32 | 2320 |
| | Cingulate Gyrus | -4 | -4 | 38 | 1288 |
| | Cingulate Gyrus | 6 | -12 | 40 | 1376 |
| | Cingulate Gyrus | 14 | -26 | 42 | 752 |
| | Cingulate Gyrus | 6 | 8 | 42 | 3272 |
| | Cingulate Gyrus | -8 | 8 | 44 | 1888 |
| | Cingulate Gyrus | 16 | -34 | 48 | 536 |
| | Cingulate Gyrus | -6 | -12 | 44 | 1128 |
| | Cingulate Gyrus | 2 | -4 | 48 | 2696 |
| | Cingulate Gyrus | -10 | -4 | 48 | 1088 |
| | Medial Frontal Gyrus | 8 | 16 | 50 | 1544 |

| | | | | | |
|---|---|---|---|---|---|
| | Medial Frontal Gyrus | 4 | -10 | 58 | 1256 |
| | Medial Frontal Gyrus | 14 | -4 | 58 | 656 |
| | Medial Frontal Gyrus | -4 | -8 | 64 | 1112 |
| | Medial Frontal Gyrus | 10 | 0 | 66 | 2312 |
| | Superior Frontal Gyrus | 8 | 8 | 58 | 2360 |
| | Superior Frontal Gyrus | -4 | 2 | 62 | 2952 |
| | Superior Frontal Gyrus | -12 | -6 | 66 | 864 |
| Cluster 7 | Middle Frontal Gyrus | -34 | 40 | 28 | 3232 |
| | Middle Frontal Gyrus | -34 | 40 | 22 | 632 |
| | Middle Frontal Gyrus | -32 | 38 | 28 | 1144 |
| | Middle Frontal Gyrus | -36 | 44 | 30 | 1456 |
| Cluster 8 | Precentral Gyrus | -40 | -14 | 40 | 104 |
| Cluster 9 | Middle Frontal Gyrus | 50 | 0 | 44 | 2584 |
| | Middle Frontal Gyrus | 52 | 2 | 42 | 1280 |
| | Precentral Gyrus | 48 | -2 | 48 | 1304 |
| Cluster 10 | Paracentral Lobule | -20 | -38 | 56 | 2792 |
| | Cingulate Gyrus | -16 | -32 | 42 | 680 |
| | Paracentral Lobule | -16 | -42 | 50 | 368 |
| | Postcentral Gyrus | -22 | -32 | 58 | 928 |
| | Postcentral Gyrus | -22 | -40 | 64 | 816 |
| Cluster 11 | Postcentral Gyrus | 22 | -30 | 56 | 384 |

Note. **Conjunction of increased activity for visceral and somatic stimulation, independent of study.** Peak statistics are reported at a threshold of $q_{FDR} < .05$. Coordinates are reported in MNI space.

Table S7. Conjunction of brain regions exhibiting decreased activation during somatic and visceral stimulation versus rest.

| Cluster | Peak region | x | y | z | Volume ($mm^3$) |
|---|---|---|---|---|---|
| Cluster 1 | Superior Temporal Gyrus | 48 | 14 | -34 | 384 |
| | Middle Temporal Gyrus | 50 | 12 | -34 | 208 |
| | Superior Temporal Gyrus | 46 | 16 | -34 | 176 |
| Cluster 2 | Middle Temporal Gyrus | -56 | 0 | -28 | 488 |
| | Middle Temporal Gyrus | -52 | 2 | -32 | 176 |
| | Middle Temporal Gyrus | -60 | 0 | -24 | 312 |
| Cluster 3 | Middle Temporal Gyrus | -46 | 10 | -32 | 504 |
| | Middle Temporal Gyrus | -48 | 10 | -32 | 168 |
| | Superior Temporal Gyrus | -36 | 10 | -32 | 184 |
| | Superior Temporal Gyrus | -56 | 12 | -26 | 152 |
| Cluster 4 | Parahippocampal Gyrus | 28 | -16 | -22 | 456 |
| Cluster 5 | Parahippocampal Gyrus | -26 | -16 | -22 | 448 |
| Cluster 6 | Medial Frontal Gyrus | 6 | 10 | -22 | 120 |
| Cluster 7 | Parahippocampal Gyrus | 32 | -30 | -18 | 192 |
| | Parahippocampal Gyrus | 28 | -30 | -20 | 96 |
| | Parahippocampal Gyrus | 36 | -32 | -18 | 96 |
| Cluster 8 | Middle Temporal Gyrus | -60 | -12 | -16 | 1368 |
| | Inferior Temporal Gyrus | -58 | -10 | -18 | 752 |
| | Middle Temporal Gyrus | -62 | -6 | -16 | 112 |
| | Middle Temporal Gyrus | -58 | -20 | -16 | 184 |
| | Middle Temporal Gyrus | -60 | -16 | -12 | 320 |
| Cluster 9 | Inferior Temporal Gyrus | 62 | -8 | -16 | 592 |
| | Inferior Temporal Gyrus | 58 | -8 | -18 | 264 |
| | Middle Temporal Gyrus | 64 | -10 | -12 | 328 |
| Cluster 10 | Medial Frontal Gyrus | -12 | 48 | 16 | 35376 |
| | Anterior Cingulate | 8 | 38 | -12 | 1024 |
| | Anterior Cingulate | 4 | 26 | -10 | 832 |
| | Anterior Cingulate | -6 | 26 | -10 | 528 |
| | Anterior Cingulate | -2 | 34 | -6 | 872 |
| | Anterior Cingulate | 6 | 38 | -6 | 1072 |
| | Anterior Cingulate | -12 | 44 | 0 | 416 |
| | Medial Frontal Gyrus | -10 | 46 | -18 | 400 |
| | Medial Frontal Gyrus | 0 | 54 | -10 | 928 |
| | Medial Frontal Gyrus | -10 | 38 | -10 | 928 |

| | | | | | |
|---|---|---|---|---|---|
| | Medial Frontal Gyrus | -4 | 46 | -8 | 1776 |
| | Medial Frontal Gyrus | 10 | 50 | -8 | 808 |
| | Medial Frontal Gyrus | -10 | 52 | -4 | 832 |
| | Medial Frontal Gyrus | -6 | 52 | 4 | 1440 |
| | Medial Frontal Gyrus | 6 | 56 | 2 | 1016 |
| | Medial Frontal Gyrus | -2 | 64 | 2 | 1360 |
| | Medial Frontal Gyrus | -2 | 58 | 10 | 2008 |
| | Medial Frontal Gyrus | 2 | 66 | 14 | 288 |
| | Medial Frontal Gyrus | -10 | 60 | 14 | 1720 |
| | Medial Frontal Gyrus | 4 | 52 | 16 | 936 |
| | Medial Frontal Gyrus | 8 | 60 | 20 | 944 |
| | Medial Frontal Gyrus | -6 | 54 | 24 | 1400 |
| | Middle Frontal Gyrus | -30 | 20 | 42 | 888 |
| | Middle Frontal Gyrus | -20 | 28 | 48 | 2040 |
| | Middle Frontal Gyrus | -24 | 20 | 50 | 2072 |
| | Middle Frontal Gyrus | -34 | 20 | 52 | 1736 |
| | Subcallosal Gyrus | -6 | 18 | -16 | 96 |
| | Subcallosal Gyrus | -6 | 24 | -16 | 192 |
| | Superior Frontal Gyrus | -14 | 56 | 32 | 1704 |
| | Superior Frontal Gyrus | -10 | 48 | 38 | 1056 |
| | Superior Frontal Gyrus | -20 | 38 | 40 | 880 |
| | Superior Frontal Gyrus | -12 | 38 | 50 | 1272 |
| | Superior Frontal Gyrus | -20 | 38 | 50 | 1912 |
| Cluster 11 | Parahippocampal Gyrus | -30 | -36 | -14 | 432 |
| Cluster 12 | Posterior Cingulate | 0 | -52 | 24 | 12192 |
| | Cingulate Gyrus | -4 | -46 | 34 | 2432 |
| | Parahippocampal Gyrus | -14 | -48 | 6 | 360 |
| | Posterior Cingulate | -10 | -54 | 12 | 968 |
| | Posterior Cingulate | 14 | -50 | 10 | 640 |
| | Posterior Cingulate | 8 | -56 | 16 | 936 |
| | Posterior Cingulate | -4 | -56 | 24 | 4176 |
| | Precuneus | 8 | -54 | 28 | 2424 |
| | Thalamus | 22 | -34 | 6 | 256 |
| Cluster 13 | Middle Occipital Gyrus | 46 | -68 | 28 | 10928 |
| | Cuneus | 32 | -88 | 24 | 88 |
| | Cuneus | 30 | -86 | 30 | 312 |
| | Middle Occipital Gyrus | 40 | -80 | 30 | 728 |

| | | | | | |
|---|---|---|---|---|---|
| | Middle Occipital Gyrus | 36 | -76 | 40 | 1256 |
| | Middle Temporal Gyrus | 54 | -64 | 18 | 1032 |
| | Middle Temporal Gyrus | 48 | -56 | 18 | 1072 |
| | Middle Temporal Gyrus | 50 | -74 | 20 | 312 |
| | Middle Temporal Gyrus | 42 | -60 | 22 | 656 |
| | Middle Temporal Gyrus | 48 | -66 | 30 | 3456 |
| | Superior Occipital Gyrus | 24 | -84 | 42 | 680 |
| | Superior Temporal Gyrus | 58 | -58 | 24 | 1336 |
| Cluster 14 | Middle Occipital Gyrus | -40 | -72 | 32 | 11016 |
| | Angular Gyrus | -50 | -70 | 32 | 824 |
| | Angular Gyrus | -46 | -66 | 36 | 1080 |
| | Cuneus | -30 | -90 | 28 | 128 |
| | Cuneus | -22 | -90 | 32 | 376 |
| | Middle Occipital Gyrus | -38 | -72 | 36 | 1672 |
| | Middle Occipital Gyrus | -28 | -82 | 38 | 1536 |
| | Middle Temporal Gyrus | -46 | -64 | 18 | 680 |
| | Middle Temporal Gyrus | -44 | -62 | 24 | 2376 |
| | Superior Occipital Gyrus | -38 | -78 | 28 | 832 |
| | Superior Parietal Lobule | -26 | -78 | 48 | 720 |
| | Superior Temporal Gyrus | -56 | -60 | 24 | 728 |
| Cluster 15 | Middle Frontal Gyrus | 24 | 30 | 46 | 6936 |
| | Middle Frontal Gyrus | 28 | 22 | 42 | 1128 |
| | Middle Frontal Gyrus | 22 | 30 | 40 | 904 |
| | Middle Frontal Gyrus | 26 | 36 | 46 | 888 |
| | Middle Frontal Gyrus | 28 | 20 | 52 | 1352 |
| | Superior Frontal Gyrus | 12 | 54 | 38 | 320 |
| | Superior Frontal Gyrus | 18 | 38 | 46 | 704 |
| | Superior Frontal Gyrus | 26 | 30 | 52 | 1640 |
| Cluster 16 | Precentral Gyrus | -40 | -16 | 68 | 1064 |
| | Precentral Gyrus | -48 | -16 | 62 | 184 |
| | Precentral Gyrus | -38 | -22 | 70 | 312 |
| | Precentral Gyrus | -36 | -14 | 68 | 528 |
| Custer 17 | Superior Parietal Lobule | 20 | -68 | 62 | 704 |
| | Superior Parietal Lobule | 18 | -72 | 62 | 296 |
| | Superior Parietal Lobule | 22 | -66 | 62 | 408 |
| Cluster 18 | Superior Parietal Lobule | -20 | -68 | 64 | 416 |
| | Superior Parietal Lobule | -16 | -72 | 64 | 152 |

| | | | | | |
|---|---|---|---|---|---|
| | Superior Parietal Lobule | -22 | -66 | 64 | 264 |
| Cluster 19 | Postcentral Gyrus | -46 | -32 | 66 | 248 |
| | Postcentral Gyrus | -48 | -28 | 64 | 128 |
| | Postcentral Gyrus | -42 | -34 | 68 | 120 |

Note. **Conjunction of decreased activity for visceral and somatic stimulation, independent of study.** The first entry for each cluster denotes the peak coordinate and volume of the entire cluster; subsequent entries for each cluster denote peak coordinates and volumes of sub-clusters. Peak statistics are reported at a threshold of $q_{FDR} < .05$. Coordinates are reported in MNI space.

Table S8. Mean point-biserial correlation (Cohen's *d*, p-value) between brain response to stimulation versus rest and resting-state networks for each study.

| Study | Visual | Somatomotor | Dorsal Attention | Ventral Attention | Limbic | Fronto-parietal | Default |
|---|---|---|---|---|---|---|---|
| Study 1 (gastric pain) | -0.048 ( -0.083, .305) | -0.007 ( -0.021, .794) | 0.001 (0.005, 0.951) | *0.078 (0.333, .001)* | -0.053 ( -0.148, .079) | *0.051 (0.370, <.0001)* | *-0.065 (-0.232, .010)* |
| Study 2 (rectal discomfort 1) | -0.079 (-0.198, .023) | *-0.097 (-0.269, .004)* | *-0.065 (-0.332, .001)* | *0.089 (0.363, <.0001)* | 0.001 (0.006, 0.943) | 0.087 (0.006, 0.004) | -0.038 (-0.119, .149) |
| Study 3 (rectal discomfort 2) | -0.028 (-0.083, .295) | *-0.082 (-0.298, .001)* | *-0.084 (-0.392, <.0001)* | *0.096 (0.500, <.0001)* | -0.023 (-0.100, 0.211) | *0.047 (0.253, 0.003)* | *-0.055 (-0.193, .019)* |
| Study 4 (esophageal pain) | 0.027 (0.101, .203) | *0.105 (0.524, <.0001)* | 0.008 (0.071, 0.371) | *0.246 (1.34, <.0001)* | *-0.085 (-0.627, <.0001)* | *0.049 (0.194, 0.018)* | *-0.241 (-0.905, <.0001)* |
| Study 5 (vulvar pressure) | -0.037 (-0.095, .240) | *0.102 (0.262, .005)* | -0.035 (-0.109, 0.185) | *0.174 (0.706, <.0001)* | -0.027 (-0.098, 0.229) | 0.019 (0.066, 0.412) | *-0.060 (-0.202, .021)* |
| Study 6 (thermal pain 1) | *-0.160 (-0.454, <.0001)* | *0.158 (0.510, <.0001)* | *-0.096 (-0.483, <.0001)* | *0.205 (0.984, <.0001)* | *-0.023 (-0.152, 0.061)* | -0.041 (-0.162, 0.048) | *-0.138 (-0.572, <.0001)* |
| Study 7 (thermal pain 2) | -0.025 (-0.092, .245) | *0.101 (0.471, <.0001)* | -0.009 (-0.064, 0.414) | *0.142 (1.03, <.0001)* | *-0.049 (-0.252, 0.003)* | -0.037 (-0.160, 0.049) | *-0.148 (-0.694, <.0001)* |

Note. P-values are reported in parenthesis and are based on a one-sample t-test of fisher-transformed correlation coefficients. *P*-values less than 0.023 survive the false discovery rate threshold $q_{FDR} < .05$ (italic).

Table S9. Brain regions exhibiting increased activity during stimulation versus rest (conjunction somatic – visceral) and greater amplitude of activation for somatic compared to visceral stimulation.

| Cluster | Peak region | x | y | z | Volume ($mm^3$) | Peak t – statistic |
|---|---|---|---|---|---|---|
| Cluster 1 | Thalamus | 8 | -16 | -2 | 190240 | 13.35 |
| | Caudate | -18 | 28 | 6 | 256 | 4.50 |
| | Caudate | -16 | 4 | 24 | 360 | 4.28 |
| | Caudate | 20 | -16 | 22 | 328 | 7.39 |
| | Caudate | 18 | -2 | 24 | 336 | 4.46 |
| | Cerebellar Lingual | -6 | -48 | -20 | 8400 | 7.18 |
| | Cerebellum | -2 | -84 | -30 | 2152 | 3.90 |
| | Cerebellum | -22 | -38 | -30 | 3472 | 6.11 |
| | Claustrum | 26 | 28 | 0 | 720 | 4.75 |
| | Claustrum | -28 | 28 | -2 | 976 | 5.01 |
| | Claustrum | 38 | -18 | 12 | 8600 | 11.90 |
| | Claustrum | 36 | 4 | 10 | 5848 | 13.35 |
| | Claustrum | 26 | 18 | 14 | 520 | 4.97 |
| | Culmen | -24 | -54 | -30 | 3888 | 5.07 |
| | Culmen | -50 | -46 | -28 | 1832 | 5.00 |
| | Culmen | 8 | -38 | -22 | 3984 | 5.20 |
| | Declive | -28 | -68 | -28 | 2728 | 4.29 |
| | Declive | -4 | -66 | -18 | 3712 | 4.84 |
| | Fusiform Gyrus | -44 | -32 | -24 | 248 | 2.99 |
| | Inferior Frontal Gyrus | 32 | 8 | -14 | 2792 | 6.62 |
| | Inferior Parietal Lobule/ TPJ | 60 | -34 | 28 | 5368 | 8.98 |
| | Inferior Temporal Gyrus | 54 | -26 | -30 | 368 | 3.20 |
| | Inferior Temporal Gyrus | -50 | -38 | -22 | 832 | 4.29 |
| | Inferior Temporal Gyrus | 48 | -28 | -14 | 1896 | 5.33 |
| | Insula | -42 | 8 | -6 | 6264 | 8.08 |
| | Insula | 40 | -14 | -6 | 6336 | 9.23 |
| | Insula | -42 | -12 | -2 | 4312 | 5.71 |
| | Insula | 48 | 8 | 0 | 11320 | 10.58 |
| | Insula | 36 | 22 | 2 | 9376 | 9.61 |
| | Insula | -32 | 24 | 6 | 4184 | 8.04 |
| | Insula | -42 | -20 | 14 | 6152 | 6.68 |
| | Insula | -38 | 4 | 10 | 5424 | 9.16 |
| | Insula | 46 | -30 | 22 | 5008 | 9.76 |

| | | | | | | |
|---|---|---|---|---|---|---|
| | Lentiform Nucleus | 30 | -10 | -2 | 1440 | 6.62 |
| | Lentiform Nucleus | 18 | -4 | -4 | 2080 | 5.70 |
| | Lentiform Nucleus | 22 | -6 | 8 | 1752 | 5.40 |
| | Lentiform Nucleus | -22 | 16 | 14 | 576 | 5.41 |
| | Parahippocampal Gyrus | -42 | -28 | -16 | 576 | 3.43 |
| | Parahippocampal Gyrus | -36 | -42 | -2 | 312 | 3.59 |
| | Postcentral Gyrus/Parietal Operculum | 60 | -20 | 20 | 6544 | 8.45 |
| | Postcentral Gyrus/Parietal Operculum | -58 | -32 | 22 | 10320 | 7.48 |
| | Precentral Gyrus | 58 | 0 | 8 | 10256 | 10.83 |
| | Precentral Gyrus | -56 | 0 | 8 | 12096 | 10.09 |
| | Pyramis | 10 | -88 | -34 | 696 | 3.15 |
| | Red Nucleus | 0 | -28 | -6 | 2464 | 5.41 |
| | Subcallosal Gyrus | -26 | 8 | -16 | 224 | 2.89 |
| | Substantia Nigra | 10 | -20 | -10 | 4216 | 7.82 |
| | Superior Temporal Gyrus | 44 | -32 | -2 | 408 | 4.78 |
| | Thalamus | 2 | -6 | 12 | 3432 | 5.87 |
| | Transverse Temporal Gyrus | -62 | -16 | 10 | 3064 | 4.68 |
| | Tuber | -16 | -88 | -38 | 792 | 3.38 |
| | Tuber | -40 | -76 | -38 | 720 | 3.13 |
| | Tuber | -32 | -82 | -38 | 1312 | 3.40 |
| | Tuber | -48 | -68 | -36 | 584 | 3.36 |
| | Tuber | -44 | -58 | -32 | 2936 | 4.54 |
| | Tuber of Vermis | -4 | -70 | -34 | 1256 | 3.78 |
| | Uvula | -16 | -72 | -32 | 4192 | 5.81 |
| Cluster 2 | Tuber | 54 | -60 | -38 | 920 | 3.21 |
| | Tuber | 54 | -58 | -40 | 704 | 3.21 |
| | Tuber | 50 | -60 | -34 | 216 | 2.66 |
| Cluster 3 | Tuber | 40 | -82 | -36 | 1080 | 3.39 |
| Cluster 4 | Declive | 18 | -70 | -26 | 320 | 3.01 |
| Cluster 5 | Medial Frontal Gyrus | 10 | -14 | 58 | 80584 | 12.81 |
| | Caudate | 2 | 22 | 12 | 440 | 3.71 |
| | Cingulate Gyrus | -2 | 16 | 30 | 6512 | 7.74 |
| | Cingulate Gyrus | 10 | -20 | 46 | 3672 | 7.16 |
| | Cingulate Gyrus | -8 | -4 | 46 | 5256 | 8.10 |
| | Cingulate Gyrus | 4 | 2 | 46 | 11496 | 11.63 |
| | Medial Frontal Gyrus | 6 | -8 | 66 | 19656 | 8.34 |
| | Medial Frontal Gyrus | 6 | -30 | 74 | 7496 | 6.08 |

| | | | | | | |
|---|---|---|---|---|---|---|
| | Middle Frontal Gyrus | 52 | 0 | 46 | 1912 | 4.13 |
| | Postcentral Gyrus | 18 | -46 | 68 | 7064 | 12.81 |
| | Postcentral Gyrus | 8 | -50 | 76 | 1408 | 6.10 |
| | Precentral Gyrus | 36 | -18 | 52 | 1120 | 4.28 |
| | Precentral Gyrus | 16 | -30 | 62 | 5976 | 6.48 |
| | Precentral Gyrus | 30 | -30 | 66 | 8576 | 7.33 |
| Cluster 6 | Middle Frontal Gyrus | -34 | 42 | 28 | 1200 | 3.11 |
| Cluster 7 | Postcentral Gyrus | -18 | -40 | 62 | 8360 | 5.95 |
| | Cingulate Gyrus | -16 | -36 | 46 | 1168 | 4.12 |
| | Postcentral Gyrus | -18 | -34 | 62 | 2960 | 4.09 |
| | Postcentral Gyrus | -18 | -46 | 66 | 4232 | 5.95 |

Note. **Average effect of somatic versus visceral stimulation, independent of study.** The first entry for each cluster denotes the peak coordinate and volume of the entire cluster; subsequent entries for each cluster denote peak coordinates and volumes of sub-clusters. Peak statistics are reported at a threshold of $q_{FDR} < .05$. Coordinates are reported in MNI space. *No gray matter within 5mm of peak.

Table S10. Brain regions exhibiting increased activity during stimulation versus rest (conjunction somatic – visceral) and greater amplitude of activation for visceral compared to somatic stimulation.

| Cluster | Peak region | x | y | z | Volume (mm$^3$) | Peak t - statistic |
|---|---|---|---|---|---|---|
| Cluster 1 | Lingual Gyrus | 4 | -74 | 2 | 27584 | 6.82 |
| | Culmen | 36 | -48 | -26 | 192 | 4.67 |
| | Culmen | 24 | -52 | -20 | 2160 | 6.82 |
| | Cuneus | -2 | -72 | 12 | 2320 | 4.79 |
| | Fusiform Gyrus | -36 | -72 | -16 | 424 | 4.53 |
| | Lingual Gyrus | -20 | -82 | -14 | 1520 | 4.16 |
| | Lingual Gyrus | 26 | -72 | -16 | 912 | 4.07 |
| | Lingual Gyrus | -10 | -90 | -8 | 2864 | 4.57 |
| | Lingual Gyrus | -18 | -96 | -8 | 584 | 4.58 |
| | Lingual Gyrus | 12 | -84 | -6 | 4480 | 4.73 |
| | Lingual Gyrus | 0 | -78 | 2 | 5176 | 4.48 |
| | Parahippocampal Gyrus | 20 | -56 | -8 | 1312 | 4.96 |
| | Posterior Cingulate | 16 | -62 | 6 | 664 | 3.32 |
| | Posterior Cingulate | 10 | -68 | 14 | 864 | 4.19 |
| | Precuneus | 16 | -66 | 24 | 408 | 3.73 |
| | Precuneus | 4 | -74 | 26 | 1112 | 4.34 |
| | Precuneus | -4 | -74 | 34 | 776 | 3.73 |
| | Precuneus | 10 | -66 | 44 | 1248 | 5.49 |
| | Precuneus | -4 | -70 | 44 | 568 | 3.72 |
| Cluster 2 | Lentiform Nucleus | -28 | -10 | -8 | 792 | 3.66 |
| | Amygdala | -22 | -6 | -16 | 104 | 2.77 |
| | Lentiform Nucleus | -30 | -10 | -8 | 688 | 3.66 |
| Cluster 3 | Middle Frontal Gyrus | 42 | 40 | -16 | 40 | 2.67 |
| Cluster 4 | Thalamus | -18 | -26 | 0 | 1872 | 5.10 |
| | Parahippocampal Gyrus | -22 | -30 | -8 | 400 | 3.78 |
| | Thalamus | -16 | -24 | 2 | 1472 | 5.10 |
| Cluster 5 | Middle Temporal Gyrus | -54 | -56 | -2 | 1792 | 5.85 |
| | Middle Temporal Gyrus | -50 | -62 | -4 | 592 | 5.85 |
| | Middle Temporal Gyrus | -56 | -54 | -4 | 656 | 5.21 |
| | Middle Temporal Gyrus | -60 | -48 | 6 | 544 | 3.52 |
| Cluster 6 | Middle Temporal Gyrus | 58 | -48 | -6 | 776 | 4.41 |
| Cluster 7 | Thalamus | 20 | -28 | 0 | 408 | 5.80 |

| | | | | | | |
|---|---|---|---|---|---|---|
| Cluster 8 | Posterior Cingulate | 0 | -34 | 24 | 1152 | 4.04 |
| | Cingulate Gyrus | 6 | -30 | 28 | 344 | 3.30 |
| | Cingulate Gyrus | -6 | -26 | 32 | 136 | 2.78 |
| | Posterior Cingulate | 0 | -36 | 22 | 672 | 4.04 |
| Cluster 9 | Anterior Cingulate | 10 | 36 | 22 | 56 | 2.61 |
| Cluster 10 | Middle Frontal Gyrus | -40 | 0 | 44 | 4920 | 5.61 |
| | Inferior Frontal Gyrus | -50 | 6 | 36 | 2240 | 5.61 |
| | Middle Frontal Gyrus | -26 | -2 | 50 | 832 | 3.10 |
| | Middle Frontal Gyrus | -34 | 0 | 52 | 960 | 3.62 |
| | Middle/Superior Frontal Gyrus | -26 | -4 | 60 | 464 | 3.77 |
| | Precentral Gyrus | -36 | -10 | 58 | 424 | 4.08 |
| Cluster 11 | Precentral Gyrus | 36 | 8 | 40 | 1904 | 4.83 |
| | Middle Frontal Gyrus | 40 | 16 | 30 | 296 | 3.33 |
| | Middle Frontal Gyrus | 32 | 4 | 48 | 912 | 4.83 |
| | Precentral Gyrus | 40 | 10 | 36 | 696 | 3.42 |
| Cluster 12 | Precuneus | -18 | -70 | 36 | 112 | 3.46 |
| | Precuneus | -20 | -70 | 32 | 72 | 3.46 |
| | Precuneus | -18 | -68 | 40 | 40 | 2.65 |
| Cluster 13 | Middle Frontal Gyrus | 48 | 30 | 34 | 48 | 2.61 |
| Cluster 14 | Inferior Parietal Lobule | -40 | -44 | 44 | 12928 | 9.53 |
| | Inferior Parietal Lobule | -38 | -44 | 42 | 4720 | 6.53 |
| | Inferior Parietal Lobule | -48 | -34 | 46 | 4024 | 7.57 |
| | Inferior Parietal Lobule | -44 | -48 | 54 | 1624 | 6.77 |
| | Postcentral Gyrus | -60 | -20 | 40 | 448 | 3.54 |
| | Superior Parietal Lobule | -30 | -56 | 44 | 2112 | 9.53 |
| Cluster 15 | Inferior Parietal Lobule | 40 | -46 | 44 | 9064 | 6.80 |
| | Inferior Parietal Lobule | 40 | -44 | 42 | 3560 | 5.62 |
| | Inferior Parietal Lobule | 36 | -56 | 44 | 3048 | 6.80 |
| | Inferior Parietal Lobule | 50 | -40 | 50 | 2456 | 4.84 |
| Cluster 16 | Middle Frontal Gyrus | -44 | 30 | 40 | 192 | 4.84 |
| Cluster 17 | Cingulate Gyrus | 12 | -40 | 42 | 72 | 3.61 |
| Cluster 18 | Cingulate Gyrus | -10 | -44 | 44 | 56 | 3.12 |
| Cluster 19 | Superior Frontal Gyrus | -20 | 14 | 66 | 8 | 2.62 |

Note. **Average effect of visceral versus somatic stimulation, independent of study.** The first entry for each cluster denotes the peak coordinate and volume of the entire cluster; subsequent entries for each cluster denote peak coordinates and volumes of sub-clusters. Peak statistics are reported at a threshold of $q_{FDR} < .05$. Coordinates are reported in MNI space.

Table S11. Brain regions exhibiting decreased activity during stimulation versus rest (conjunction somatic – visceral) and greater deactivation amplitude for somatic compared to visceral stimulation.

| Cluster | Peak region | x | y | z | Volume ($mm^3$) | Peak t - statistic |
|---|---|---|---|---|---|---|
| Cluster 1 | Superior Temporal Gyrus | 46 | 16 | -34 | 888 | 3.94 |
| | Superior Temporal Gyrus | 36 | 14 | -34 | 256 | 2.74 |
| | Superior Temporal Gyrus | 50 | 16 | -34 | 632 | 3.94 |
| Cluster 2 | Middle Temporal Gyrus | -56 | 2 | -34 | 104 | 2.82 |
| Cluster 3 | Superior Temporal Gyrus | -36 | 10 | -34 | 96 | 2.56 |
| Cluster 4 | Middle Temporal Gyrus | -56 | 12 | -28 | 408 | 3.36 |
| | Middle Temporal Gyrus | -48 | 12 | -34 | 56 | 2.71 |
| | Middle Temporal Gyrus | -56 | 12 | -28 | 352 | 3.36 |
| Cluster 5 | Posterior Cingulate | -6 | -52 | 24 | 273752 | 9.73 |
| | Cingulate Gyrus | -6 | -44 | 32 | 6744 | 8.90 |
| | Cingulate Gyrus | 6 | -46 | 40 | 9152 | 9.61 |
| | Culmen | 26 | -48 | -16 | 5488 | 7.25 |
| | Cuneus | -24 | -94 | -4 | 4560 | 4.89 |
| | Cuneus | 0 | -76 | 6 | 3656 | 5.05 |
| | Fusiform Gyrus | 32 | -36 | -20 | 4136 | 8.26 |
| | Fusiform Gyrus | -36 | -50 | -14 | 2392 | 7.31 |
| | Fusiform Gyrus | 42 | -58 | -12 | 5704 | 7.13 |
| | Inferior Frontal Gyrus | -50 | 24 | 24 | 2392 | 4.23 |
| | Inferior Occipital Gyrus | -34 | -84 | -12 | 4720 | 5.75 |
| | Inferior Occipital Gyrus | 40 | -76 | -10 | 6008 | 6.87 |
| | Inferior Occipital Gyrus | -12 | -90 | -10 | 2616 | 4.57 |
| | Inferior Parietal Lobule | -46 | -58 | 42 | 8480 | 6.23 |
| | Inferior Parietal Lobule | -36 | -44 | 44 | 5560 | 6.53 |
| | Inferior Parietal Lobule | 38 | -44 | 44 | 4488 | 5.62 |
| | Inferior Parietal Lobule | 50 | -56 | 44 | 5032 | 4.77 |
| | Inferior Parietal Lobule | -46 | -34 | 48 | 6008 | 7.57 |
| | Inferior Parietal Lobule | 50 | -34 | 50 | 2392 | 4.67 |
| | Inferior Temporal Gyrus | -42 | -64 | -10 | 3920 | 8.25 |
| | Inferior Temporal Gyrus | -44 | -76 | -4 | 4960 | 8.21 |
| | Lingual Gyrus | 26 | -82 | -10 | 4072 | 4.91 |
| | Lingual Gyrus | 12 | -86 | -6 | 3376 | 4.73 |
| | Middle Frontal Gyrus | -44 | 8 | 40 | 6152 | 5.79 |

| | | | | | |
|---|---|---|---|---|---|
| Middle Frontal Gyrus | -44 | 20 | 38 | 4856 | 6.66 |
| Middle Frontal Gyrus | -20 | 32 | 48 | 904 | 3.73 |
| Middle Frontal Gyrus | -38 | 20 | 50 | 2392 | 5.07 |
| Middle Frontal Gyrus | -36 | 8 | 56 | 3896 | 6.00 |
| Middle Occipital Gyrus | 26 | -96 | 2 | 2936 | 5.84 |
| Middle Occipital Gyrus | 38 | -86 | 8 | 5704 | 5.39 |
| Middle Occipital Gyrus | -32 | -88 | 14 | 6888 | 5.53 |
| Middle Temporal Gyrus | -56 | -4 | -22 | 2088 | 4.20 |
| Middle Temporal Gyrus | -50 | -56 | -12 | 2280 | 7.66 |
| Middle Temporal Gyrus | -64 | -8 | -16 | 592 | 3.19 |
| Middle Temporal Gyrus | -58 | -26 | -8 | 3360 | 4.16 |
| Middle Temporal Gyrus | -60 | -44 | -4 | 3128 | 4.71 |
| Middle Temporal Gyrus | 46 | -58 | 22 | 4936 | 4.41 |
| Middle Temporal Gyrus | 44 | -78 | 26 | 3264 | 5.57 |
| Parahippocampal Gyrus | -22 | -12 | -20 | 2248 | 3.93 |
| Parahippocampal Gyrus | 26 | -14 | -20 | 1544 | 3.94 |
| Parahippocampal Gyrus | 16 | -6 | -22 | 200 | 3.09 |
| Parahippocampal Gyrus | -28 | -36 | -16 | 2392 | 7.05 |
| Postcentral Gyrus | -52 | -22 | 54 | 1768 | 5.52 |
| Postcentral Gyrus | -42 | -28 | 62 | 3256 | 5.04 |
| Posterior Cingulate | 12 | -50 | 8 | 7328 | 6.13 |
| Posterior Cingulate | -6 | -56 | 12 | 6272 | 6.37 |
| Precentral Gyrus | -32 | -16 | 66 | 4000 | 4.44 |
| Precuneus | 4 | -66 | 24 | 7544 | 7.48 |
| Precuneus | 34 | -74 | 30 | 6704 | 6.30 |
| Precuneus | 0 | -58 | 34 | 6608 | 7.43 |
| Precuneus | -28 | -76 | 32 | 8080 | 5.53 |
| Precuneus | -28 | -74 | 52 | 5456 | 7.33 |
| Precuneus | 20 | -62 | 50 | 7040 | 6.75 |
| Precuneus | 22 | -76 | 52 | 2536 | 6.56 |
| Sub-Gyral | 54 | -50 | -10 | 1152 | 4.79 |
| Superior Frontal Gyrus | -16 | 60 | 30 | 640 | 4.25 |
| Superior Frontal Gyrus | -18 | 50 | 42 | 824 | 3.67 |
| Superior Frontal Gyrus | -14 | 38 | 52 | 1544 | 4.11 |
| Superior Frontal Gyrus | -22 | 24 | 56 | 4360 | 4.96 |
| Superior Parietal Lobule | 36 | -76 | 42 | 3104 | 5.94 |
| Superior Parietal Lobule | -32 | -60 | 52 | 8792 | 9.73 |

| | | | | | | |
|---|---|---|---|---|---|---|
| | Superior Parietal Lobule | 34 | -62 | 52 | 6840 | 7.65 |
| | Superior Temporal Gyrus | -50 | -60 | 26 | 5704 | 4.55 |
| | Superior Temporal Gyrus | 54 | -64 | 28 | 3272 | 4.80 |
| | Thalamus | -16 | -28 | 0 | 2296 | 5.10 |
| | Thalamus | 22 | -34 | 0 | 2856 | 6.44 |
| | Uncus | -28 | -6 | -26 | 160 | 2.90 |
| Cluster 6 | Anterior Cingulate | -2 | 46 | -2 | 20248 | 4.37 |
| | Anterior Cingulate | 6 | 32 | -10 | 1376 | 3.33 |
| | Anterior Cingulate | -6 | 40 | -6 | 2160 | 3.72 |
| | Anterior Cingulate | 6 | 46 | -2 | 3904 | 4.37 |
| | Anterior Cingulate | -6 | 52 | 0 | 2872 | 4.13 |
| | Inferior Frontal Gyrus | -12 | 42 | -20 | 384 | 2.88 |
| | Medial Frontal Gyrus | 8 | 10 | -24 | 256 | 3.55 |
| | Medial Frontal Gyrus | -2 | 68 | 4 | 1872 | 3.72 |
| | Medial Frontal Gyrus | -4 | 58 | 12 | 2136 | 3.91 |
| | Medial Frontal Gyrus | 4 | 56 | 14 | 352 | 2.79 |
| | Medial Frontal Gyrus | -6 | 44 | 20 | 1408 | 3.11 |
| | Medial Frontal Gyrus | 10 | 42 | 22 | 216 | 3.08 |
| | Medial Frontal Gyrus | -6 | 42 | 30 | 296 | 2.92 |
| | Middle Frontal Gyrus | -18 | 42 | -20 | 392 | 3.14 |
| | Rectal Gyrus | -10 | 26 | -24 | 656 | 3.79 |
| | Subcallosal Gyrus | -4 | 14 | -14 | 336 | 2.89 |
| | Subcallosal Gyrus | -10 | 24 | -14 | 1632 | 3.31 |
| Cluster 7 | Medial Frontal Gyrus | -6 | 62 | -18 | 312 | 2.92 |
| Cluster 8 | Middle Temporal Gyrus | 62 | -12 | -14 | 2240 | 3.96 |
| | Middle Temporal Gyrus | 66 | -10 | -14 | 952 | 3.96 |
| | Middle Temporal Gyrus | 58 | -12 | -14 | 1000 | 3.74 |
| | Middle Temporal Gyrus | 70 | -16 | -12 | 288 | 3.59 |
| Cluster 9 | Superior Frontal Gyrus | 38 | 46 | -18 | 144 | 3.45 |
| Cluster 10 | Middle Frontal Gyrus | -34 | 50 | -10 | 448 | 2.93 |
| Cluster 11 | Inferior Frontal Gyrus | -50 | 34 | -8 | 1280 | 3.57 |
| Cluster 12 | Superior Frontal Gyrus | -26 | 56 | 6 | 160 | 2.68 |
| Cluster 13 | Middle Frontal Gyrus | 32 | 18 | 50 | 13832 | 5.37 |
| | Middle Frontal Gyrus | 26 | 32 | 42 | 976 | 2.99 |
| | Middle Frontal Gyrus | 30 | 12 | 48 | 3896 | 5.34 |
| | Middle Frontal Gyrus | 28 | 30 | 52 | 800 | 3.64 |
| | Middle Frontal Gyrus | 30 | 16 | 58 | 3328 | 5.37 |

| | | | | | | |
|---|---|---|---|---|---|---|
| | Precentral Gyrus | 38 | 22 | 42 | 4208 | 3.88 |
| | Superior Frontal Gyrus | 22 | 26 | 56 | 624 | 3.68 |
| Cluster 14 | Superior Frontal Gyrus | 16 | 56 | 38 | 296 | 3.38 |

Note. **Average effect of somatic versus visceral stimulation, independent of study.** The first entry for each cluster denotes the peak coordinate and volume of the entire cluster; subsequent entries for each cluster denote peak coordinates and volumes of sub-clusters. Peak statistics are reported at a threshold of $q_{FDR} < .05$. Coordinates are reported in MNI space. *No gray matter within 5mm of peak.

Table S12. Brain regions exhibiting decreased activity during stimulation versus rest (conjunction somatic – visceral) and greater deactivation amplitude for visceral compared to somatic stimulation.

| Cluster | Peak region | x | y | z | Cluster volume ($mm^3$) | Peak t - statistic |
|---|---|---|---|---|---|---|
| Cluster 1 | Middle Temporal Gyrus | 52 | -48 | -40 | 8 | 2.59 |
| Cluster 2 | Culmen | -22 | -26 | -32 | 40 | 3.98 |
| Cluster 3 | Fusiform/Inferior Temporal Gyrus | -46 | -34 | -26 | 72 | 2.77 |
| Cluster 4 | Fusiform/Inferior Temporal Gyrus | 56 | -22 | -28 | 16 | 2.66 |
| Cluster 5 | Parahippocampal/Fusiform Gyrus | -32 | -44 | -2 | 616 | 3.59 |
| | Parahippocampal/Fusiform Gyrus | -36 | -38 | -8 | 128 | 3.47 |
| | Parahippocampal/Fusiform Gyrus | -32 | -44 | -2 | 488 | 3.59 |
| Cluster 6 | Parahippocampal Gyrus | 36 | -42 | -2 | 200 | 4.00 |
| | Hippocampus | 34 | -46 | 0 | 72 | 3.44 |
| | Parahippocampal Gyrus | 36 | -38 | -4 | 128 | 4.00 |
| Cluster 7 | Caudate Head | 8 | 26 | 2 | 464 | 4.15 |
| | Caudate Head | 16 | 30 | 0 | 176 | 4.15 |
| | Caudate Head | 2 | 24 | 4 | 288 | 3.34 |
| Cluster 8 | Caudate Head | -16 | 30 | 2 | 240 | 4.50 |
| | Caudate Head | -10 | 26 | 4 | 48 | 3.05 |
| Cluster 9 | Caudate Tail | -20 | -38 | 14 | 80 | 3.68 |
| Cluster 10 | Caudate Body | -20 | -18 | 26 | 144 | 3.22 |
| Cluster 11 | Precentral Gyrus | 20 | -24 | 76 | 9328 | 5.89 |
| | Medial Frontal Gyrus | 4 | -32 | 76 | 1960 | 5.78 |
| | Medial Frontal Gyrus | 6 | -20 | 76 | 1040 | 5.89 |
| | Medial Frontal Gyrus | -6 | -22 | 78 | 512 | 4.04 |
| | Precentral Gyrus | 36 | -22 | 54 | 2256 | 4.63 |
| | Precentral Gyrus | 34 | -28 | 70 | 1160 | 5.68 |
| | Precentral Gyrus | 16 | -26 | 78 | 920 | 4.89 |
| | Precuneus | 6 | -46 | 78 | 304 | 4.93 |
| | Superior Frontal Gyrus | 20 | -12 | 76 | 1176 | 5.76 |
| Cluster 12 | Medial Frontal Gyrus | 6 | -24 | 60 | 1536 | 5.77 |
| | Medial Frontal Gyrus | 6 | -26 | 60 | 1008 | 4.59 |
| | Medial Frontal Gyrus | 6 | -22 | 60 | 528 | 5.77 |
| Cluster 13 | Superior Parietal Lobule | 24 | -54 | 72 | 192 | 5.84 |
| | Superior Parietal Lobule | 30 | -48 | 70 | 32 | 5.26 |
| | Superior Parietal Lobule | 18 | -58 | 72 | 64 | 3.19 |

| | | | | | | |
|---|---|---|---|---|---|---|
| | Superior Parietal Lobule | 26 | -52 | 72 | 96 | 5.84 |
| Cluster 14 | Superior Parietal Lobule | 6 | -54 | 70 | 40 | 4.07 |

Note. Average effect of visceral versus somatic stimulation, masked within regions showing negative effects of stimulation, independent of study. The first entry for each cluster denotes the peak coordinate and volume of the entire cluster; subsequent entries for each cluster denote peak coordinates and volumes of sub-clusters. Peak statistics are reported at a threshold of $q_{FDR} < .05$. Coordinates are reported in MNI space. *No gray matter within 5mm of peak.

## Supplementary References

1. Rubio, A., *et al.* Uncertainty in anticipation of uncomfortable rectal distension is modulated by the autonomic nervous system — A fMRI study in healthy volunteers. *NeuroImage* **107**, 10-22 (2015).

2. Kano, M., *et al.* Influence of Uncertain Anticipation on Brain Responses to Aversive Rectal Distension in Patients With Irritable Bowel Syndrome. *Psychosom. Med.* **79**, 988-999 (2017).

3. Pazmany, E., *et al.* Brain responses to vestibular pain and its anticipation in women with Genito-Pelvic Pain/Penetration Disorder. *NeuroImage: Clinical* **16**, 477-490 (2017).

4. Coen, S.J., *et al.* Neuroticism Influences Brain Activity During the Experience of Visceral Pain. *Gastroenterology* **141**, 909-917.e901 (2011).

5. Krishnan, A., *et al.* Somatic and vicarious pain are represented by dissociable multivariate brain patterns. *eLife* **5**, e15166 (2016).

6. Wager , T.D., *et al.* An fMRI-Based Neurologic Signature of Physical Pain. *New England Journal of Medicine* **368**, 1388-1397 (2013).

7. Dunckley, P., *et al.* Cortical processing of visceral and somatic stimulation: Differentiating pain intensity from unpleasantness. *Neuroscience* **133**, 533-542 (2005).

8. Payne, K., *et al.* Effects of Sexual Arousal on Genital and Non-Genital Sensation: A Comparison of Women with Vulvar Vestibulitis Syndrome and Healthy Controls. *Arch. Sex. Behav.* **36**, 289-300 (2007).

9. Pukall, C.F., Binik, Y.M. & Khalife, S. A New Instrument for Pain Assessment in Vulvar Vestibulitis Syndrome. *Journal of Sex and Marital Therapy* **30**, 69-78 (2004).

10. Pukall, C.F., *et al.* Neural correlates of painful genital touch in women with vulvar vestibulitis syndrome. *Pain* **115**, 118-127 (2005).

11. Pukall, C.F., Young, R.A., Roberts, M.J., Sutton, K.S. & Smith, K.B. The vulvalgesiometer as a device to measure genital pressure-pain threshold. *Physiol. Meas.* **28**, 1543-1550 (2007).

12. Schweinhardt, P., Kuchinad, A., Pukall, C.F. & Bushnell, M.C. Increased gray matter density in young women with chronic vulvar pain. *Pain* **140**, 411-419 (2008).

13. Yeo, B.T., *et al.* The organization of the human cerebral cortex estimated by intrinsic functional connectivity. *Journal of Neurophysiology* **106**, 1125-1165 (2011).
