# Supplementary material for: Common and distinct neural representations of aversive somatic and visceral stimulation in healthy individuals
Source: Nat Commun. 2020 Nov 23;11:5939. doi: 10.1038/s41467-020-19688-8 (PMC7684294; doi:10.1038/s41467-020-19688-8)
Supplement: Supplementary file 3 — Reporting Summary [file 41467_2020_19688_MOESM3_ESM.pdf]

# nature research

Corresponding author(s): Wager

Last updated by author(s): Sep 14, 2020

# Reporting Summary

Nature Research wishes to improve the reproducibility of the work that we publish. This form provides structure for consistency and transparency in reporting. For further information on Nature Research policies, see our Editorial Policies and the Editorial Policy Checklist.

## Statistics

For all statistical analyses, confirm that the following items are present in the figure legend, table legend, main text, or Methods section.

| n/a | Confirmed | |
|---|---|---|
| ☐ | ☒ | The exact sample size (*n*) for each experimental group/condition, given as a discrete number and unit of measurement |
| ☐ | ☒ | A statement on whether measurements were taken from distinct samples or whether the same sample was measured repeatedly |
| ☐ | ☒ | The statistical test(s) used AND whether they are one- or two-sided<br>*Only common tests should be described solely by name; describe more complex techniques in the Methods section.* |
| ☐ | ☒ | A description of all covariates tested |
| ☐ | ☒ | A description of any assumptions or corrections, such as tests of normality and adjustment for multiple comparisons |
| ☐ | ☒ | A full description of the statistical parameters including central tendency (e.g. means) or other basic estimates (e.g. regression coefficient) AND variation (e.g. standard deviation) or associated estimates of uncertainty (e.g. confidence intervals) |
| ☐ | ☒ | For null hypothesis testing, the test statistic (e.g. $F$, $t$, $r$) with confidence intervals, effect sizes, degrees of freedom and $P$ value noted<br>*Give P values as exact values whenever suitable.* |
| ☒ | ☐ | For Bayesian analysis, information on the choice of priors and Markov chain Monte Carlo settings |
| ☐ | ☒ | For hierarchical and complex designs, identification of the appropriate level for tests and full reporting of outcomes |
| ☐ | ☒ | Estimates of effect sizes (e.g. Cohen's *d*, Pearson's *r*), indicating how they were calculated |

*Our web collection on statistics for biologists contains articles on many of the points above.*

## Software and code

Policy information about availability of computer code

| | |
|---|---|
| Data collection | Stimulus presentation was conducted using different software, depending on the particular study. Software languages include Presentation, Visual Basic, E-prime, and MATLAB code -- we cannot verify the version number of software packages used. |
| Data analysis | Preprocessing and first level modeling of fMRI data was performed using the SPM toolbox for MATLAB (SPM8 and SPM12 -- https://www.fil.ion.ucl.ac.uk/spm/). Second level regression and classification models were implemented using the CANLab core tools (https://github.com/canlab). |

For manuscripts utilizing custom algorithms or software that are central to the research but not yet described in published literature, software must be made available to editors and reviewers. We strongly encourage code deposition in a community repository (e.g. GitHub). See the Nature Research guidelines for submitting code & software for further information.

## Data

Policy information about availability of data

All manuscripts must include a data availability statement. This statement should provide the following information, where applicable:

- Accession codes, unique identifiers, or web links for publicly available datasets
- A list of figures that have associated raw data
- A description of any restrictions on data availability

Data are available from the authors on request and at https://neurovault.org/collections/8708/. Data used to validate the NPS are available at https://neurovault.org/collections/3324/.

# Field-specific reporting

Please select the one below that is the best fit for your research. If you are not sure, read the appropriate sections before making your selection.

- [x] Life sciences
- [ ] Behavioural & social sciences
- [ ] Ecological, evolutionary & environmental sciences

For a reference copy of the document with all sections, see nature.com/documents/nr-reporting-summary-flat.pdf

# Life sciences study design

All studies must disclose on these points even when the disclosure is negative.

| | |
|---|---|
| Sample size | This work is a mega-analysis of data from 7 individual studies with a total sample size (165 participants). Datasets that were individually adequately powered (produced large, reliable effects) were pooled together. |
| Data exclusions | No data were excluded from analysis. |
| Replication | Cross-validation (and testing in independent samples) showed that effects replicated across individuals and studies. |
| Randomization | Participants were randomly sampled for each study. Study covariates were included in regression models to control for study-specific effects. Hold-out tests using data from independent studies were used to verify generalization across studies. |
| Blinding | Because this work is a mega-analysis of multiple studies, experimenters were not aware of the goal to compare and contrasts different experimental manipulations (i.e., different kinds of somatic and visceral stimulation), although they were aware of group assignment. |

# Reporting for specific materials, systems and methods

We require information from authors about some types of materials, experimental systems and methods used in many studies. Here, indicate whether each material, system or method listed is relevant to your study. If you are not sure if a list item applies to your research, read the appropriate section before selecting a response.

## Materials & experimental systems

| n/a | Involved in the study | |
|---|---|---|
| x | | Antibodies |
| x | | Eukaryotic cell lines |
| x | | Palaeontology and archaeology |
| x | | Animals and other organisms |
| | x | Human research participants |
| x | | Clinical data |
| x | | Dual use research of concern |

## Methods

| n/a | Involved in the study | |
|---|---|---|
| x | | ChIP-seq |
| x | | Flow cytometry |
| | x | MRI-based neuroimaging |

## Human research participants

Policy information about studies involving human research participants

| | |
|---|---|
| Population characteristics | Study 1: N=15 (10 female), Age=31.9 ± 8.8 (SD); Study 2: N=15 (9 female), Age=29.5 ± 10.5 (SD); Study 3: N=29 (15 female) Age=22.5 ± 2.8 (SD); Study 4: N=15 (15 female), Age=23.2 ± 1.6 (SD); Study 5: N=30 (14 female), Age=30.4 ± 8.7 (SD); Study 6: N=28 (10 female), Age=25.2 ± 7.4 (SD); Study 7: N=33 (22 female), Age=27.9 ± 9.0 (SD) |
| Recruitment | Healthy volunteers were recruited by means of local advertisement, without any medical or psychiatric disorders and not currently taking any medication affecting the central nervous system were included. We do not expect self-selection biases to substantially influence results. |
| Ethics oversight | All studies were approved by the respective medical ethics committees of the institutions where the studies were performed (Study 1 and 4: University of Leuven, Belgium; Study 2: Université de Grenoble Joseph Fourier, France; Study 3: Tohoku University, Sendai, Japan; Study 5: King's College London, UK; Study 6: University of Colorado, Boulder, USA; Study 7: Columbia University, New York, USA). All subjects provided written informed consent prior to being included in the studies at all sites. |

Note that full information on the approval of the study protocol must also be provided in the manuscript.

# Magnetic resonance imaging

## Experimental design

| | |
|---|---|
| Design type | Task based fMRI |
| Design specifications | Studies 1-4: 72 trials, 30 second stimulation, 24 second inter-trial interval; Study 5: 20 trials, 1 second stimulation, 9-15 second inter-trial interval; Study 6: 81 trials, 11 second stimulation, 5-11 second jitter; Study 7: 55 trials, 12.5 second stimulation, 23-27 seconds of rest |
| Behavioral performance measures | Pain/unpleasantness self-report. The mean difference between conditions (high vs. low levels of stimulation) was of interest. |

## Acquisition

| | |
|---|---|
| Imaging type(s) | functional |
| Field strength | 3.0 Tesla (all studies) |
| Sequence & imaging parameters | Studies 1/4: EPI sequence with blood oxygen level-dependent (BOLD) contrast 980 (TR/TE=3000/30 ms, voxel size=2.50 × 2.50 × 2.50 mm^3, flip angle 90°, 48 slices of 2.5 mm thick) covering the whole brain including the cerebellum. Study 2: EPI sequence (TR/TE=3000/30 ms; flip angle 80°; 52 slices of 3 mm thick; voxel size 988 2.75 × 2.75 × 3 mm3) covering the whole brain including the cerebellum. Study 3: EPI sequence with BOLD contrast (TR/TE = 3000/30 ms, voxel size = 2.5 × 2.5 × 2.5 mm3, flip angle 90°, 50 slices) covering the whole brain including the cerebellum. Study 5: T EPI sequence (40×3-mm slices, 0.3 interslice gap, TE 30 ms, TR 2,500 1000 ms, flip angle 80°, matrix size 64^2, voxel size 3.4 × 3.4 × 3.4 mm3 voxels, 26 interleaved slices with ascending acquisition, parallel imaging with an iPAT acceleration of 2). Study 7: EPI sequence, TR = 2000 ms, TE = 20 ms, field of view = 224 mm, 64 × 64 matrix, 3 × 3 × 3 mm3 voxels, 42 interleaved slices, parallel imaging, SENSE factor 1.5 covering the whole brain. |
| Area of acquisition | Whole brain |

Diffusion MRI ☐ Used ☒ Not used

## Preprocessing

| | |
|---|---|
| Preprocessing software | SPM8 |
| Normalization | Nonlinear deformation based on anatomical data. |
| Normalization template | ICBM152 space |
| Noise and artifact removal | First level models included motion parameter estimates. |
| Volume censoring | For study 6 only, outlier timepoints were identified based on standard deviation of time-series |

## Statistical modeling & inference

| | |
|---|---|
| Model type and settings | First level models were mass-univariate, second level analyses include regression and classification models. |
| Effect(s) tested | GLM-based regression, modeling effects of stimulation type. Classification of different stimulation types. |

Specify type of analysis: ☒ Whole brain ☐ ROI-based ☐ Both

| | |
|---|---|
| Statistic type for inference (See Eklund et al. 2016) | voxel-wise |
| Correction | FDR |

## Models & analysis

| n/a | Involved in the study |
|---|---|
| ☒ | ☐ Functional and/or effective connectivity |
| ☒ | ☐ Graph analysis |
| ☐ | ☒ Multivariate modeling or predictive analysis |

| | |
|---|---|
| Multivariate modeling and predictive analysis | Classification model discriminating between somatic and visceral stimulation. Features included activation of different resting state networks. Sensitivity, specificity, AUC, and Cohen's d were estimated using crossvalidation and in independent studies. |
